# Histone and DNA methylation control by H3 serine 10/threonine 11 phosphorylation in the mouse zygote

**DOI:** 10.1186/s13072-017-0112-x

**Published:** 2017-02-14

**Authors:** Jie Lan, Konstantin Lepikhov, Pascal Giehr, Joern Walter

**Affiliations:** 1 FR 8.3, Biological Sciences, Genetics/Epigenetics, University of Saarland, Campus A2.4, 66123 Saarbrücken, Germany; 2 Faculty of Medicine, Free University of Brussels, C.P. 614, Building GE, 5th floor, 808 Route de Lennik, 1070 Brussels, Belgium

## Abstract

**Background:**

In the mammalian zygote, epigenetic reprogramming is a tightly controlled process of coordinated alterations of histone and DNA modifications. The parental genomes of the zygote show distinct patterns of histone H3 variants and distinct patterns of DNA and histone modifications. The molecular mechanisms linking histone variant-specific modifications and DNA methylation reprogramming during the first cell cycle remain to be clarified.

**Results:**

Here, we show that the degree and distribution of H3K9me2 and of DNA modifications (5mC/5hmC) are influenced by the phosphorylation status of H3S10 and H3T11. The overexpression of the mutated histone variants H3.1 and 3.2 at either serine 10 or threonine 11 causes a decrease in H3K9me2 and 5mC and a concomitant increase in 5hmC in the maternal genome. Bisulphite sequencing results indicate an increase in hemimethylated CpG positions following H3.1T10A overexpression suggesting an impact of H3S10 and H3T11 phosphorylation on DNA methylation maintenance.

**Conclusions:**

Our data suggest a crosstalk between the cell-cycle-dependent control of S10 and T11 phosphorylation of histone variants H3.1 and H3.2 and the maintenance of the heterochromatic mark H3K9me2. This histone H3 “phospho-methylation switch” also influences the oxidative control of DNA methylation in the mouse zygote.

**Electronic supplementary material:**

The online version of this article (doi:10.1186/s13072-017-0112-x) contains supplementary material, which is available to authorized users.

## Background

The epigenetic reprogramming in mouse zygote involves an extensive rearrangement of the epigenetic landscape, including chromatin reorganization and comprehensive changes in DNA modifications. These changes require a coordinated control of epigenetic “writers”, “readers”, “erasers” and “remodelers” on the level of histones and DNA after fertilization. The interplay between histone variants, chromatin modifications and DNA modifications has been studied to a great detail. Here, we analyse the synergetic dynamics of different post-translational modifications in histone H3 variants H3.1, H3.2 and H3.3 which are found in different epigenetic compartments of chromatin [1]. In mouse zygotes, these histone variants show an asymmetrical deposition into parental pronuclei: H3.3 is a predominant histone variant in the newly formed paternal pronucleus, while H3.1 and H3.2 only appear during the first replication of the paternal chromatin. In contrast, the maternal chromatin is initially enriched for H3.1/H3.2 and accumulates H3.3 at later zygotic stages [2–4]. This asymmetry in histone variant composition is accompanied by an asymmetric allocation of histone modifications in both pronuclei [2, 5, 6]. While the paternal chromatin is mainly marked by open chromatin modifications such as H3K4me3, the maternal chromatin shows a high abundance of H3K9me2 heterochromatic mark, which is slightly reduced during the first cell division [5, 7]. In pre-replicative paternal chromatin, H3K9me2 is almost absent and only becomes detectable at late replication stages. In both pronuclei, the abundance of H3K9me2 is linked to differences in DNA modifications. The H3K9me2 containing maternal pronucleus maintains 5mC as the predominant modification, and the level of DNA methylation decreases only slightly during the first DNA replication [8]. It has been shown that the presence of H3K9me2 in the maternal pronuclei protects against Tet3-mediated oxidation of 5mC to 5hmC [9]. As a consequence of this, the maternal chromosomes appear to maintain 5mC levels in contrast to the more oxidized paternal chromosomes, which are practically devoid of H3K9me2 at early stages of DNA replication and where 5mC is extensively converted to 5hmC by Tet3, reducing DNA methylations by about 50% at the end of the first cell cycle [10].

The current knowledge suggests that H3K9me2 has an important protective role for the maintenance of 5mC. Work by Nakamura et al. showed that Stella protein while present in both pronuclei only protects the maternal DNA against Tet3 oxidation due to the presence of H3K9me2 [9, 11]. However, previous data also suggest that this epigenetic control could be linked to the asymmetric distribution of the major histone variants H3.1, H3.2 and H3.3 [4]. H3S10 phosphorylation has been shown to negatively control H3K9 methylation in fruit fly [12]. In vitro biochemical assays demonstrated a protective role of H3T11 phosphorylation against H3K9me3 demethylation [13, 14]. However, interactions of H3S10phos and H3T11phos with H3K9me2 in mammalian cells have not yet been described. The vicinity of the K9, S10 and T11 residues in the N-terminus of H3 suggests a possible influence or crosstalk of modifications at these residues. This crosstalk might influence “writers” or “erasers” of individual modifications or alternatively affect the interaction with modification “readers”. Phosphorylation of histone H3 fulfils multiple roles: it participates in mitotic chromosomes condensation and segregation, but also modulates gene expression in the context-dependent manner (reviewed in [15]). Our intention was to examine the potential links between the asymmetric distribution of histone variants and the various layers of epigenetic control in both pronuclei before and after replication. In particular, we analysed the dynamics of H3S10 and H3T11 phosphorylation in the three histone H3 variants during the first cell cycle and their impact on the replication-dependent control of H3K9me2 and DNA modifications in both zygotic pronuclei. Our data reveal a direct or indirect crosstalk between H3S10 and H3T11 phosphorylation, histone variant-dependent H3K9me2 methylation and DNA methylation.

## Results

### H3S10phos and H3T11phos have different dynamics and associations with histone H3 variants in the mouse zygote

**Fig. 1:**
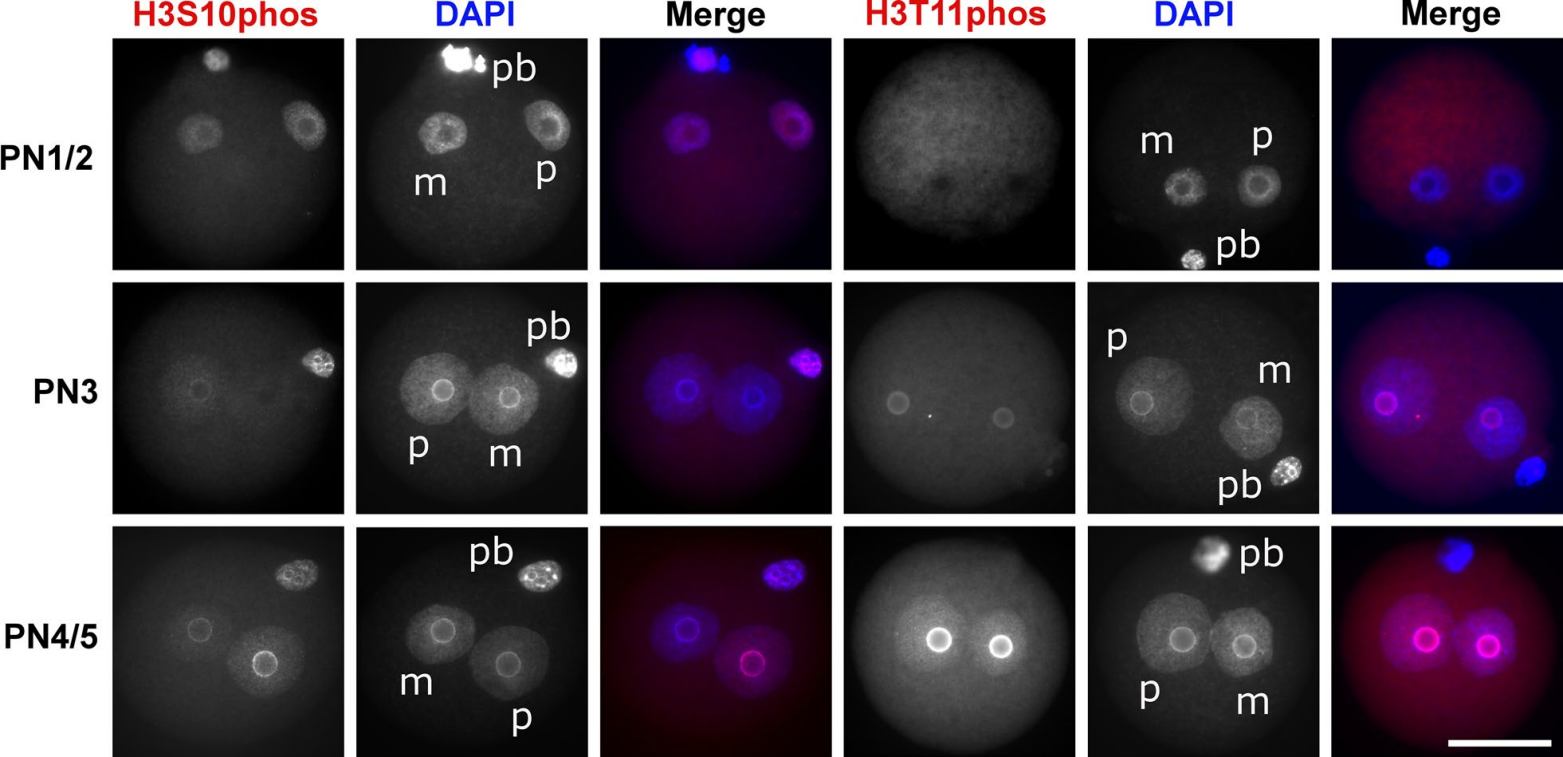
Dynamic patterns of H3S10phos and H3T11phos in mouse zygotes. Representative images of zygotes at different PN stages stained with antibodies against H3S10phos and H3T11phos, respectively. DNA is visualized by DAPI. *m* Maternal pronucleus, *p* paternal pronucleus, *pb* polar body. *Scale bar* 50 µm

We first determined the dynamics of H3S10phos and H3T11phos in the developing mouse zygotes. In line with previous reports, we observe that H3S10phos is clearly detectable in G1 (PN1/2), disappears in S (PN3) phase and reappears at late G2 (PN4/5). The mainly perinucleolar accumulation of H3S10phos is most pronounced in the paternal pronucleus at all stages [16] (Fig. 1). H3T11phos also accumulates in the perinucleolar heterochromatin but follows a different dynamic: it is absent in G1, becomes first visible during early S-phase and gradually accumulates during S-phase to remain as a strong signal up to G2 (Fig. 1). In contrast to H3S10phos which clearly shows signal intensity differences between maternal and paternal pronuclei, H3T11phos signals are equally absent or present in both pronuclei. We conclude that the neighbouring phosphorylation marks have similar nuclear patterns but different dynamics during the first cell cycle. While H3T11phos marks the replicative S and the G2 phase, H3S10phos is mainly present in the non-replicative G1 and G2 phases.

We next investigated the dynamic of H3S10 and H3T11 phosphorylation on all three histone variants. We therefore microinjected mRNAs encoding either histone wild-type H3 variants (WT) or S10A or T11A mutated forms, respectively, into early pre-replicative (2–3 h post-fertilization) mouse zygotes. The ectopically expressed wild-type or mutated forms of H3 variants were fused to GFP reporter (C-terminal fusion). This allowed us to follow their import in the pronuclei. We observe that all WT and mutant forms were readily expressed and efficiently imported into the pronuclei (Additional file 1). We assumed that the mutated non-phosphorylatable H3 variants will be incorporated into nucleosomes generating a “dominant-negative” phosphorylation effect in nucleosomes after replication at PN4/5.

**Fig. 2:**
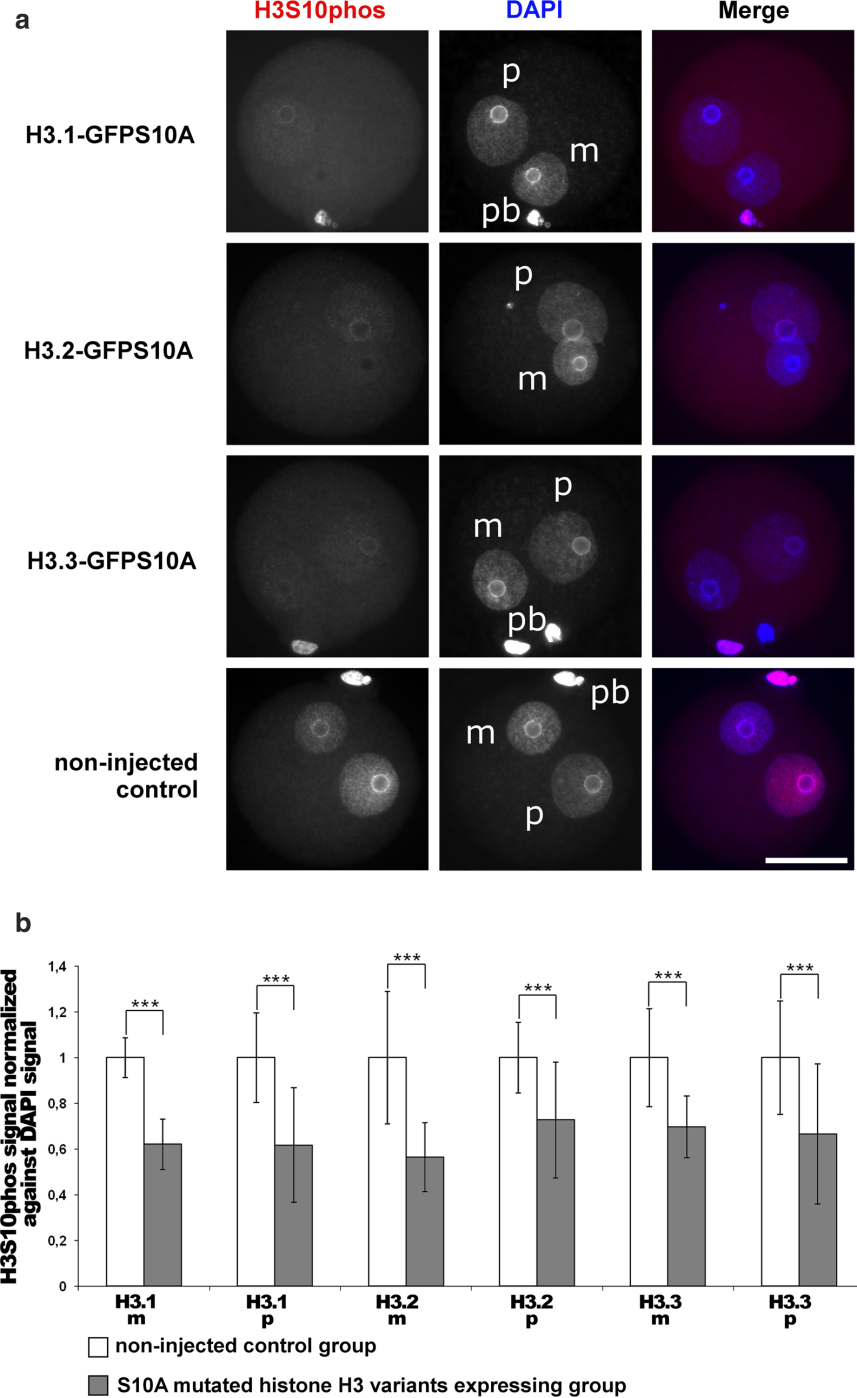
Effects of H3.1/2/3-GFPS10A expression in mouse zygotes on H3S10phos. **a** Shown are the representative images of PN4/5 stage zygotes stained with antibodies against H3S10phos. DNA is visualized by DAPI. *m* Maternal pronucleus, *p* paternal pronucleus, *pb* polar body. *Scale bar* 50 µm. **b** Quantification of H3S10phos signals, normalized against DNA signals in both parental genomes of zygotes at PN4/5. Relative signal intensities in control groups are set to 1. Statistical significance was calculated using *t* test (***P < 0.001)

**Fig. 3:**
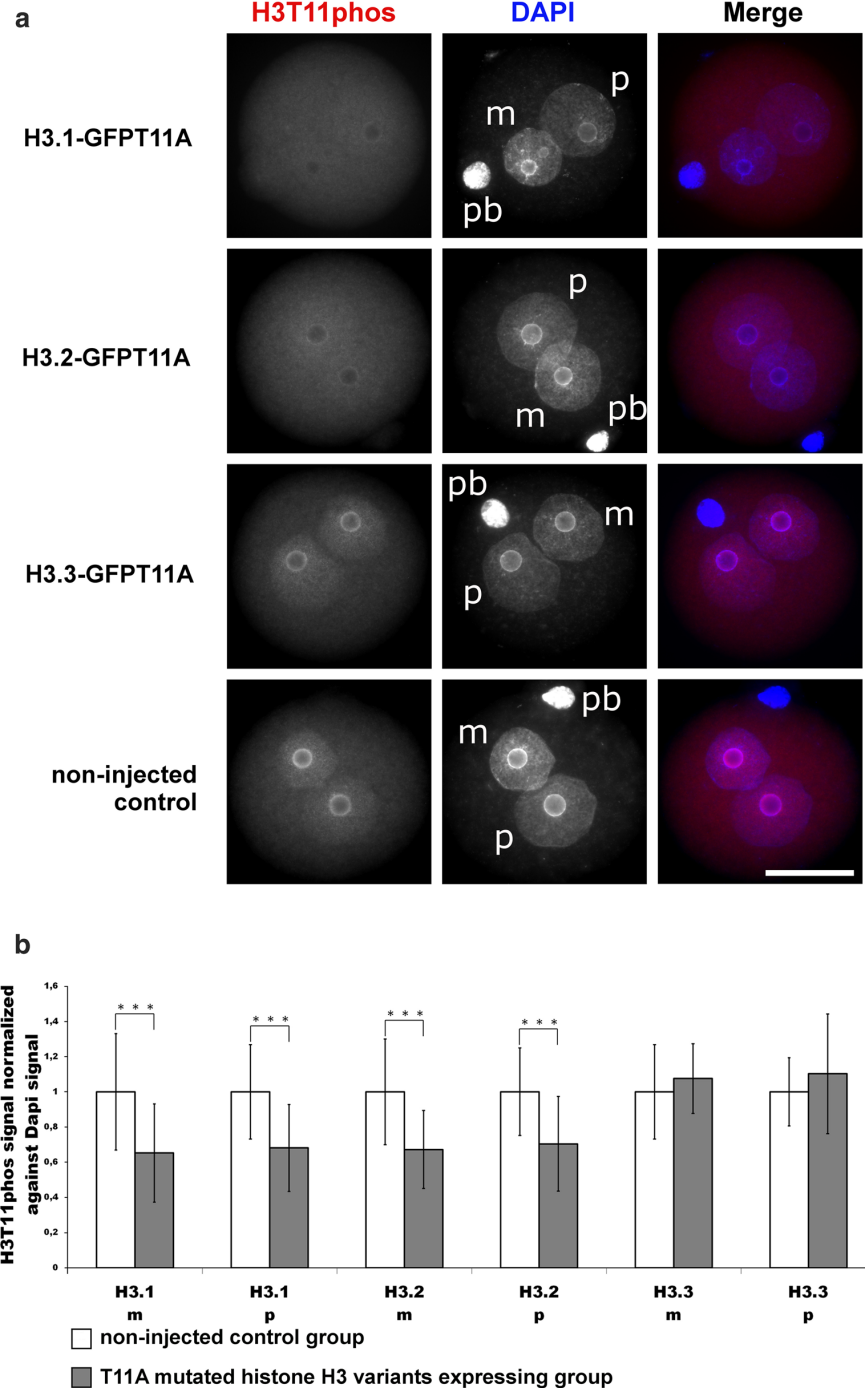
Effects of H3.1/2/3-GFPT11A expression in mouse zygotes on H3T11phos. **a** Shown are the representative images of PN4/5 stage zygotes stained with antibodies against H3T11phos. DNA is visualized by DAPI. *m* Maternal pronucleus, *p* paternal pronucleus, *pb* polar body. *Scale bar* 50 µm. **b** Quantification of H3T11phos signals, normalized against DNA signals in both parental genomes of zygotes at PN4/5. Relative signal intensities in control groups are set to 1. Statistical significance was calculated using *t* test (***P < 0.001)

Indeed, the overexpression of all three H3S10A mutated isoforms (H3.1-GFPS10A, H3.2-GFPS10A and H3.3-GFPS10A) leads to a significant decrease in H3S10phos in G2 zygotes, as visualized and measured by immunofluorescence (IF) (Fig. 2a, b). In contrast, H3T11phos was reduced when we injected and overexpressed the mutated H3.1-GFPT11A and H3.2-GFPT11A variants but remained unaffected with the H3.3-GFPT11A variant (Fig. 3a, b). Our data suggest that while all three H3 variants are equal substrates for H3S10 phosphorylation, only H3.1 and H3.2 variants are the predominant targets for H3T11 phosphorylation.

Note that the overexpression of H3WT-GFP variants in the majority of cases did not change the H3S10 and H3T11 phosphorylation pattern. However, in each experiment we observe a few examples in which overexpression leads to a reduction in the respective phosphorylation signals (Additional file 2). This effect may be caused by the time of injection or a variable amount of injected material, leading to a higher abundance of H3.1WT-GFP and H3.2WT-GFP overexpressed proteins competing with endogenous H3 for the kinase activity. Zygotes injected with H3.3WT-GFP mRNA did not show variation in H3T11phos signals (Additional file 2), supporting the notion that H3.3 is not a substrate for H3T11 phosphorylation-specific reaction.

### H3S10 and H3T11 phosphorylation is coupled to H3K9me2 histone methylation

**Fig. 4:**
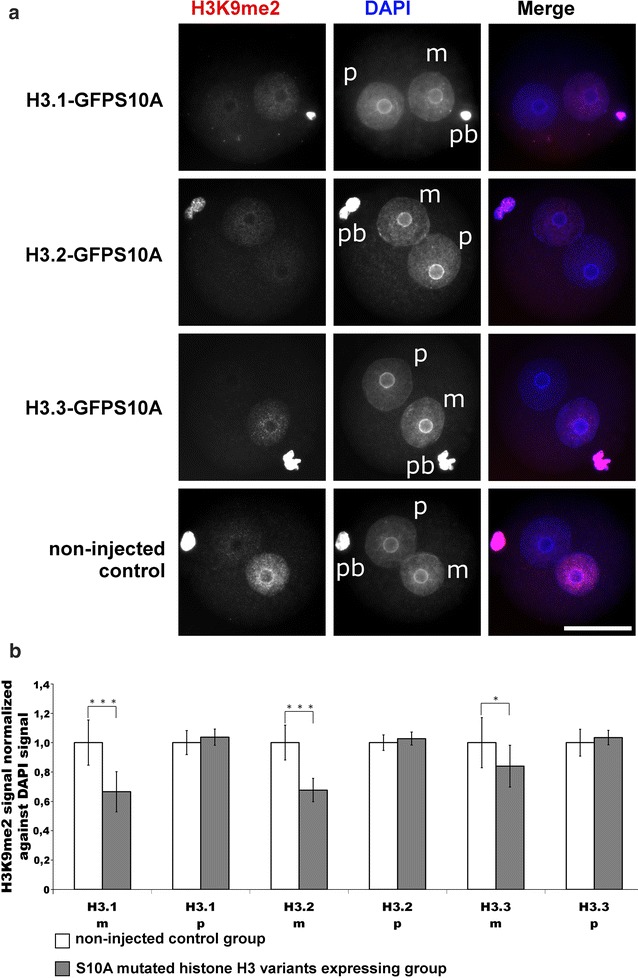
Effects of H3.1/2/3-GFPS10A expression in mouse zygotes on H3K9me2. **a** Shown are the representative images of PN4/5 stage zygotes stained with antibodies against H3K9me2. DNA is visualized by DAPI. *m* Maternal pronucleus, *p* paternal pronucleus, *pb* polar body. *Scale bar* 50 µm. **b** Quantification of H3K9me2 signals, normalized against DNA signals in both parental genomes of zygotes at PN4/5. Relative signal intensities in control groups are set to 1. Statistical significance was calculated using *t* test (***P < 0.001; *P < 0.05)

The perinucleolar heterochromatic signature of H3S10 and H3T11 phosphorylation prompted us to investigate whether: (1) phosphorylation is linked to canonical heterochromatic marks such as H3K9me2 and (2) such effects are found for all histone variants. Our approach was to individually overexpress H3S10A mutants of all three histone variants and analyse the H3K9me2 status in G2 zygotes at PN4/5. We indeed find that the overexpression of all three mutant variants caused a measurable reduction in H3K9me2 at G2 phase compared to non-injected control. A reduction in about 30% signal intensity was found in maternal pronuclei of H3.1-GFPS10A and H3.2-GFPS10A injected groups, while the H3.3-GFPS10A injected group only showed an average reduction in about 15% (Fig. 4a, b).

**Fig. 5:**
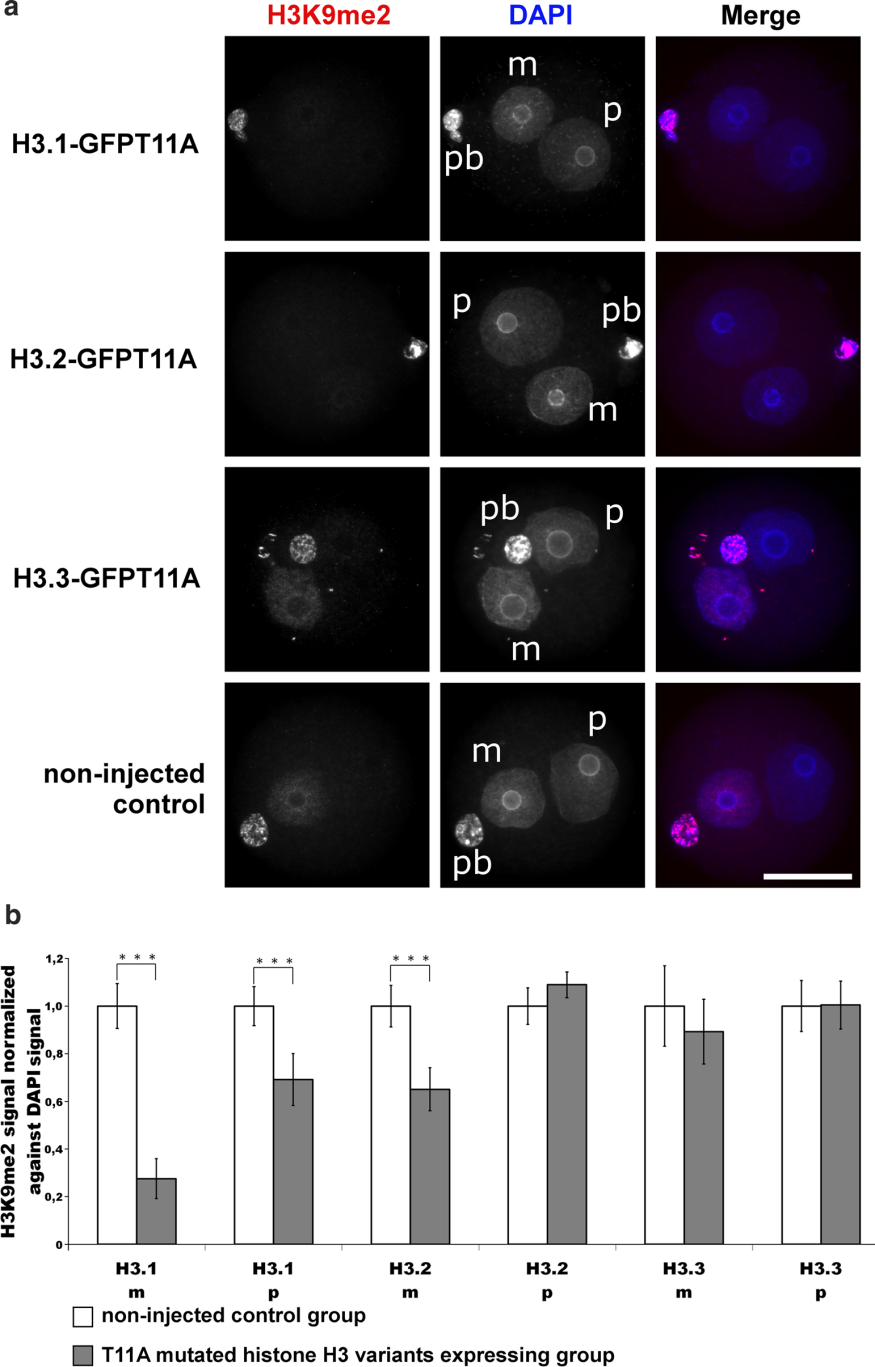
Effects of H3.1/2/3-GFPT11A expression in mouse zygotes on H3K9me2. **a** Shown are the representative images of PN4/5 stage zygotes stained with antibodies against H3K9me2. DNA is visualized by DAPI. *m* Maternal pronucleus, *p* paternal pronucleus, *pb* polar body. *Scale bar* 50 µm. **b** Quantification of H3K9me2 signals, normalized against DNA signals in both parental genomes of zygotes at PN4/5. Relative signal intensities in control groups are set to 1. Statistical significance was calculated using *t* test (***P < 0.001)

Next, we analysed H3K9me2 signals in zygotes overexpressing H3T11A mutants. We observe a strong and highly significant reduction in H3K9me2 signals in maternal pronuclei when overexpressing H3.1-GFPT11A and H3.2-GFPT11A (reduction in about 70 and 30%, respectively) but no change in both pronuclei when overexpressing H3.3-GFPT11A (Fig. 5a, b). Note that the IF signals remain constant in polar body nuclei (Figs. 4a, 5a). For H3.1-GFPT11A, we even find a mild but significant reduction in the low-level H3K9me2 in the paternal G2 pronuclei. We also performed a side-by-side comparison of zygotes, expressing H3.1-GFPT11A to zygotes expressing H3.1-GFPWT (Additional file 3). This comparison revealed a clear reduction in H3K9me2 levels in the mutants. The overexpression of WT histone H3.1 does not cause a significant reduction.

From all these experiments, we conclude that H3S10 and H3T11 phosphorylation particularly of variants H3.1 and H3.2 strongly influences the post-replicative levels of H3K9me2.

A simple explanation for a reduction in H3K9me2 signals following mutant overexpression is that the recognition and binding of anti-H3K9me2 antibody to its epitope are affected by the mutation. To examine this possibility, we co-expressed either H3.1-GFPT11A or H3.1-GFPWT together with the catalytical domain of G9a histone methyltransferase (G9aCat) in *E. coli* cells. The wild-type or mutated histones were partially purified and probed by Western blot using anti-H3K9me2 antibody. Indeed, the antibody clearly detects the H3K9me2 modification after co-expression on both WT and T11A mutated form (Additional file 4).

### H3S10 and H3T11 phosphorylation influences DNA modifications

**Fig. 6:**
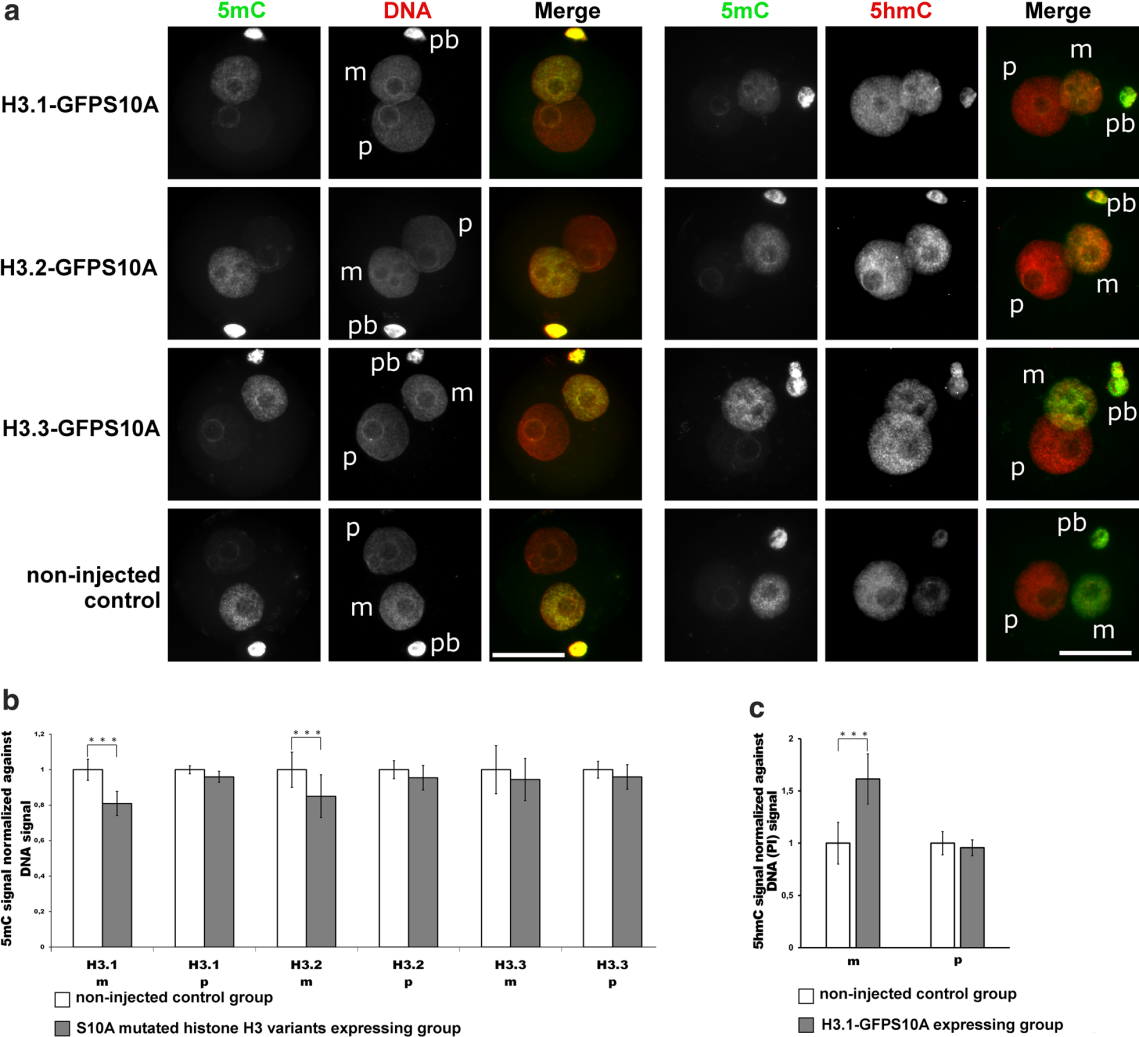
Effects of H3.1/2/3-GFPS10A expression in mouse zygotes on 5mC and 5hmC. **a** Shown are the representative images of PN4/5 stage zygotes stained with antibodies against 5mC together with anti-ssDNA antibodies, or together with 5hmC antibodies. *m* Maternal pronucleus, *p* paternal pronucleus, *pb* polar body. *Scale bar* 50 µm. **b** Quantification of 5mC signals, normalized against ssDNA signals in both parental genomes of zygotes at PN4/5. Relative signal intensities in control groups are set to 1. Statistical significance was calculated using *t* test (***P < 0.001). **c** Quantification of 5hmC signals, normalized against DNA (*PI* propidium iodide) signals in both parental genomes of zygotes at PN4/5. Relative signal intensities in control groups are set to 1. Statistical significance was calculated using *t* test (***P ≤ 0.001)

H3K9me2 has been shown to be linked to the presence of 5mC in the maternal pronucleus (reviewed in [17]). We therefore investigated whether the observed reduction in maternal H3K9me2 also affected the corresponding 5mC levels. Indeed, we find that the levels of 5mC in maternal genomes are significantly reduced by about 20–15% when overexpressing H3.1-GFPS10A and H3.2-GFPS10A mutants, respectively, while only a subtle non-significant reduction is found in H3.3-GFPS10A expressing zygotes (Fig. 6a, b). Moreover, the loss of 5mC was accompanied by a gain of 5hmC. Surprisingly, the gain of 5hmC was observed for all three H3.1, H3.2 and H3.3 S10A mutants (Fig. 6a). Hence, despite only a very subtle change in the 5mC signal in H3.3-GFPS10A expressing zygotes, the maternal pronuclei show a clear increase in 5hmC (Fig. 6a). Note that due to technical obstacles (antibody compatibility) we were unable to directly quantify the 5hmC signal, normalized against DNA antibody signal (as done for 5mC quantification). We therefore adjusted the denaturation conditions allowing us to detect anti-5hmC antibody signals and DNA signals (via propidium iodide, PI) simultaneously. Using this strategy, we were able to quantify the ratio of DNA and IF signal, and we find a clear and highly significant increase in 5hmC in the maternal pronuclei (Fig. 6c). In addition, we calculated the paternal-to-maternal ratio of 5hmC and found a significant increase in the maternal 5hmC content ratio in overexpressing zygotes suggesting a relative increase in maternal 5hmC levels (Additional file 5). Both the loss of 5mC and the gain of 5hmC in maternal pronuclei are more pronounced in H3.1-GFPS10A expressing zygotes (Fig. 6 and Additional file 5). In summary, our data suggest that the incorporation of non-phosphorylatable H3S10 variants has a variant-specific influence on the maintenance of H3K9me2 and the conversion of 5mC to 5hmC.

**Fig. 7:**
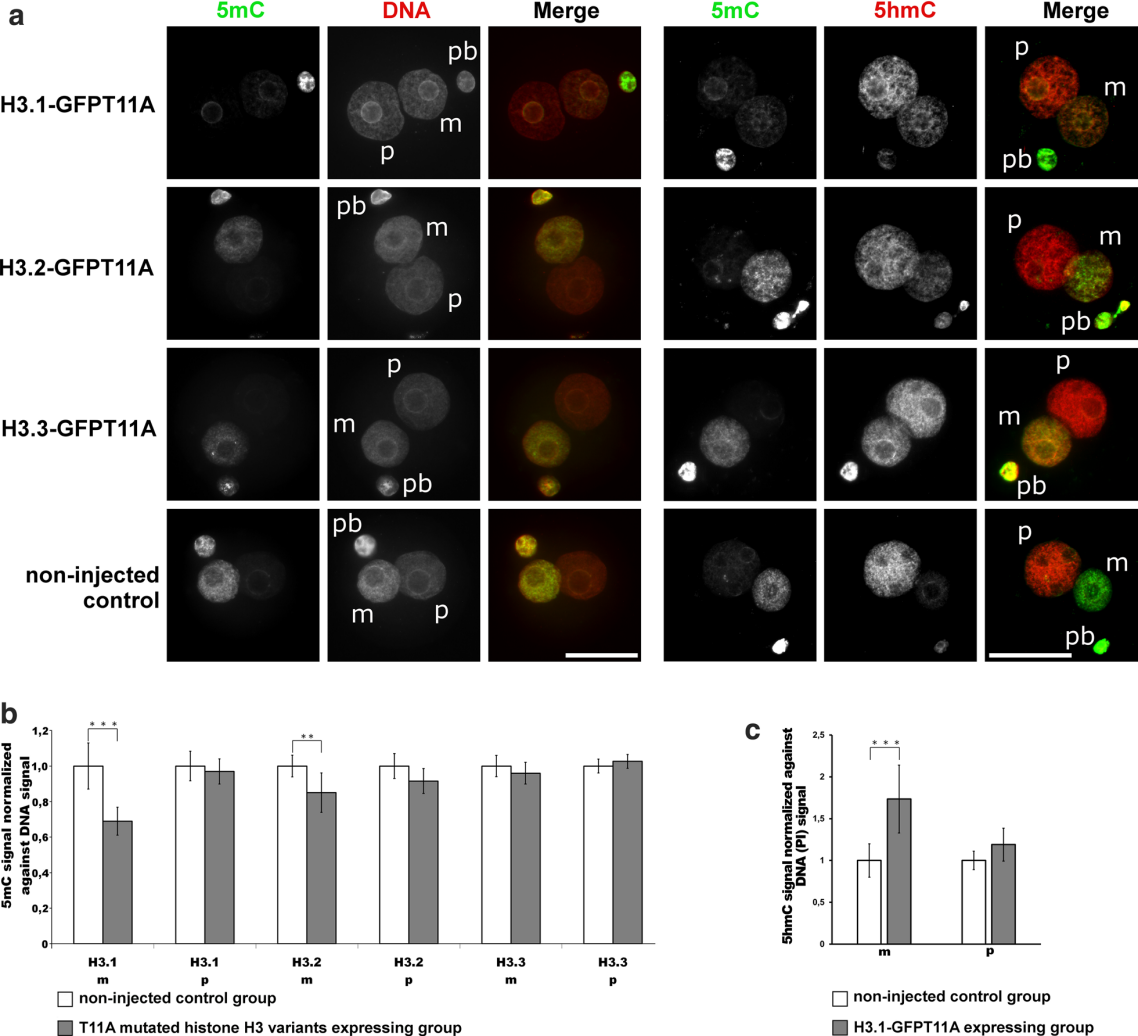
Effects of H3.1/2/3-GFPT11A expression in mouse zygotes on 5mC and 5hmC. **a** Shown are the representative images of PN4/5 stage zygotes stained with antibodies against 5mC together with anti-ssDNA antibodies, or together with 5hmC antibodies. *m* Maternal pronucleus, *p* paternal pronucleus, *pb* polar body. *Scale bar* 50 µm. **b** Quantification of 5mC signals, normalized against ssDNA signals in both parental genomes of zygotes at PN4/5. Relative signal intensities in control groups are set to 1. Statistical significance was calculated using *t* test (***P < 0.001; **P < 0.01). **c** Quantification of 5hmC signals, normalized against DNA (*PI* propidium iodide) signals in both parental genomes of zygotes at PN4/5. Relative signal intensities in control groups are set to 1. Statistical significance was calculated using *t* test (***P ≤ 0.001)

We found that overexpression of H3-GFPT11A mutants generated a very similar (almost identical) spectrum of variant-specific DNA modification changes. 5mC was significantly reduced in both H3.1- and H3.2-GFPT11A groups (Fig. 7a, b), and 5hmC signals were strongly enhanced in both H3.1-GFPT11A and H3.2-GFPT11A expressing groups compared to the controls (Fig. 7a, c and Additional file 5). Again, no significant change of 5mC was found in H3.3-GFPT11A expressing zygotes, while 5hmC in maternal pronuclei was increased in H3.3-GFPT11A expressing group (Fig. 7a).

**Fig. 8:**
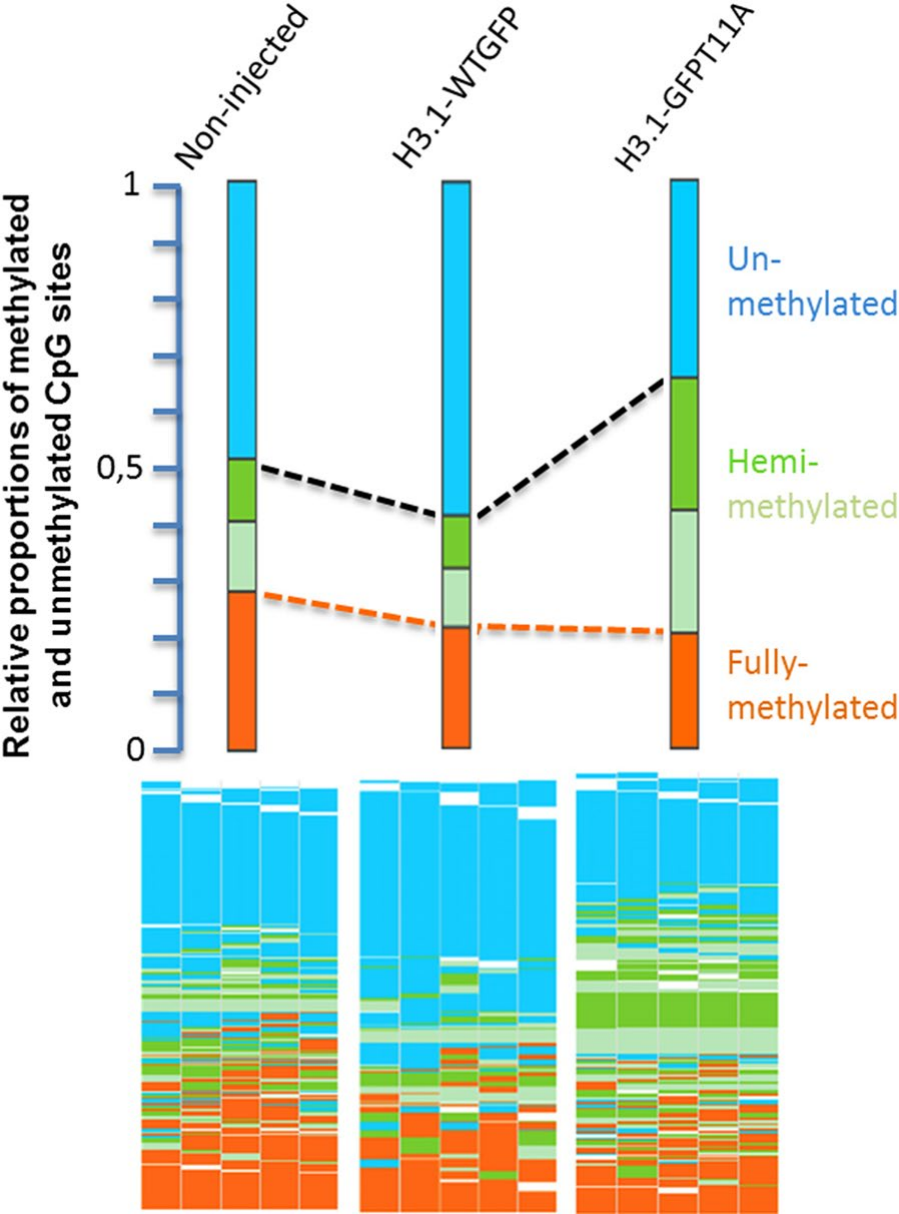
Hairpin bisulphite sequencing profiles of Line1 repetitive elements in PN4/5 zygotes expressing either H3.1-GFPWT, H3.1-GFPT11A or in non-injected control group. The *bars* represent the proportions of fully-, hemi- and unmethylated CpG dyads. The *map* below represents the distribution of methylated sites. Each *column* shows individual neighbouring CpG dyads, and each *line* represents one sequence read. The reads in the *map* are sorted according to their methylation status

Next, we examined the changes in 5hmC/5mC at the molecular level using sequencing-based approaches. We concentrated our analysis on zygotes overexpressing H3.1T11A and for which we observed the most extensive effects on 5mC and 5hmC in our IF analysis. We used hairpin bisulphite sequencing to monitor the strand-specific methylation status (methylated, unmethylated and hemimethylated) at individual CpG positions after replication [18, 19]. In contrast to the significant reduction seen in IF analysis, we only observed a small reduction in the total Line1 methylation in the H3.1-GFPT11A expressing zygotes. However, we found a strong and significant increase in hemimethylated positions in the H3.1-GFPT11A expressing group in contrast to non-injected controls (Fig. 8). This indicated that the overexpression of H3.1GFPT10A affects the DNA methylation maintenance at replication [20, 21].

### Additional H3K9me2 methylation causes only subtle changes in DNA modifications

**Fig. 9:**
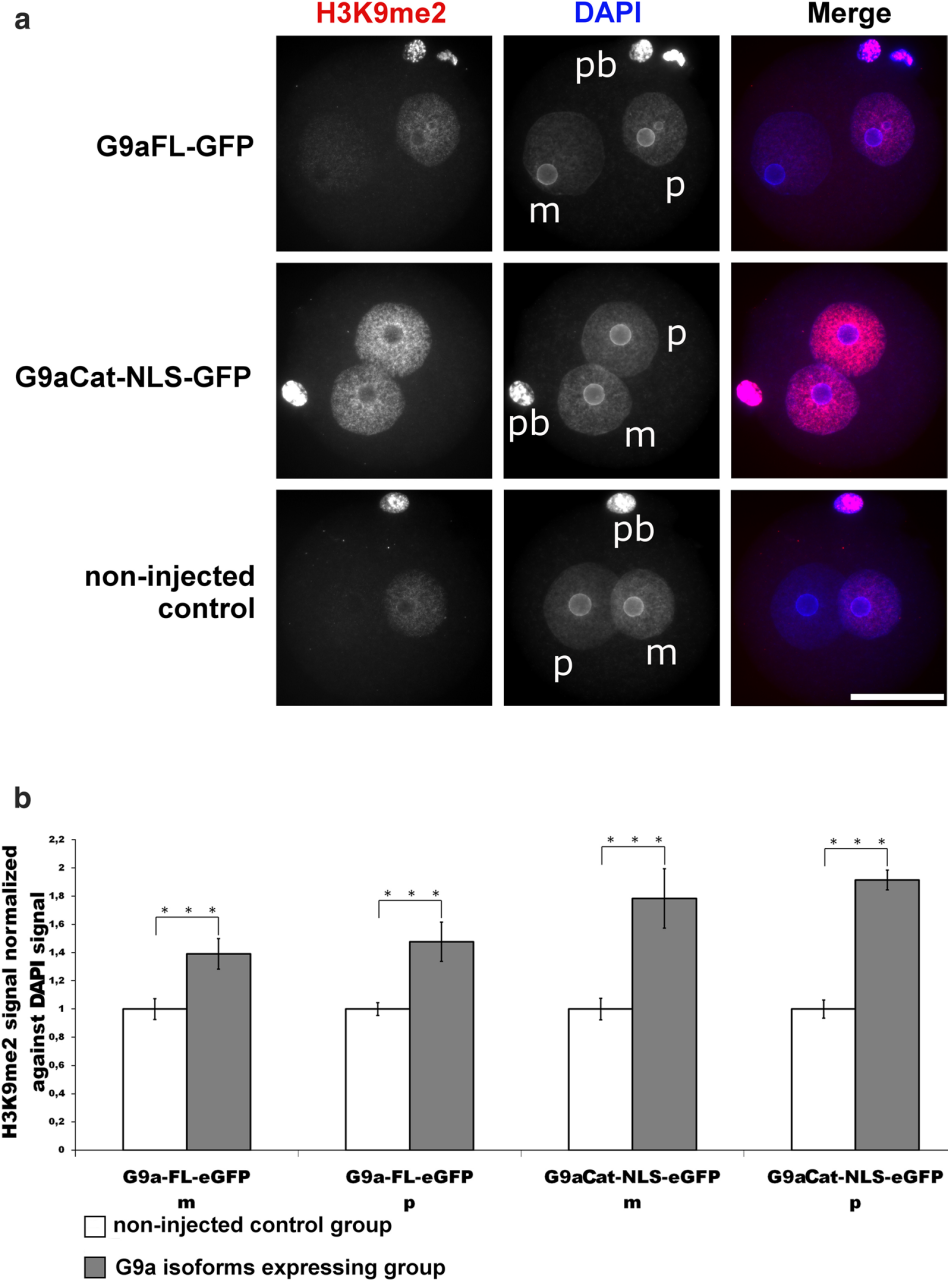
Effects of G9aFL-GFP and G9aCat-NLS-GFP expression in mouse zygotes on H3K9me2. **a** Shown are the representative images of PN4/5 stage zygotes stained with antibodies against H3K9me2. DNA is visualized by DAPI. *m* Maternal pronucleus, *p* paternal pronucleus, *pb* polar body. *Scale bar* 50 µm. **b** Quantification of H3Kme2 signals normalized against DNA signals in both parental genomes of zygotes at PN4/5. Relative signal intensities in control groups are set to 1. Statistical significance was calculated using *t* test (***P < 0.001)

H3K9me2 is not detectable on the paternal chromatin before replication and appears weakly during late replication in the mouse zygote [5, 6]. This leads us and others to the assumption that the absence of H3K9me2 in the paternal genome causes a strong Tet-mediated 5mC oxidation followed by a mostly replication-dependent “passive” demethylation [20, 21]. A correlation between H3K9me2 and 5mC/5hmC has already been shown for maternal chromatin in mouse zygotes [9]. To address the question whether an increase in H3K9me2 on paternal chromatin would influence DNA methylation and hydroxymethylation, we ectopically expressed G9a, an H3K9me2 histone methyltransferase, in mouse zygotes. We first analysed the pattern of endogenous G9a and observed its appearance (and nuclear localization) starting from the four-cell stage, but not at earlier developmental stages (Additional file 6). We first expressed a G9a full-length GFP tagged version (G9aFL-GFP) in the zygote, which lead to only a very minor effect on H3K9me2. We concluded that the N-terminus of G9a interfered with the catalytic function in the zygote, suppressing the G9a methylation function [7]. Indeed, the injection of the mRNA encoding a shorter G9aCat-NLS-GFP version overcame this control and efficiently enhanced the H3K9me2 [but not H3K9me3 (see Additional file 7)] signal in both maternal and paternal chromatin (Fig. 9).

**Fig. 10:**
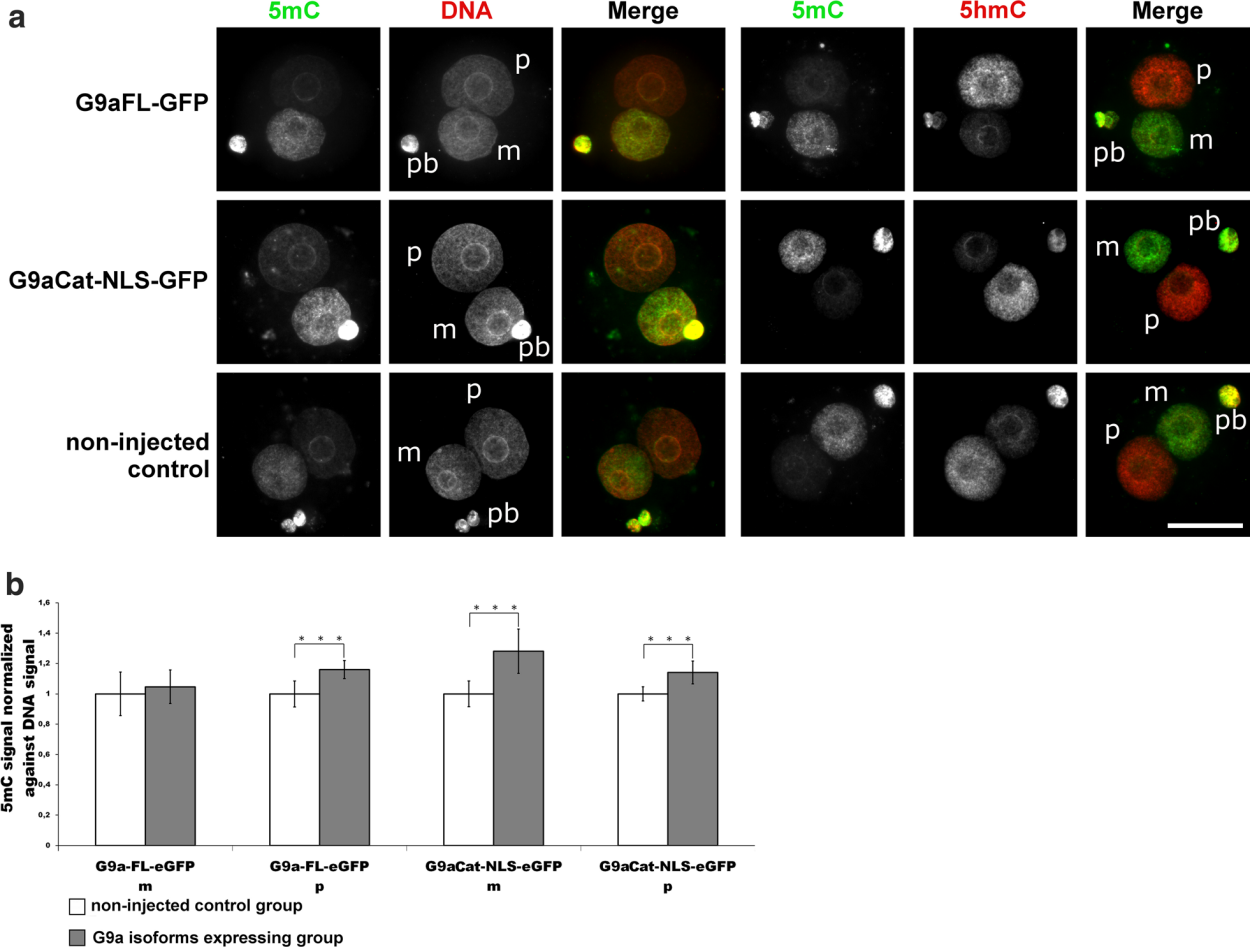
Effects of G9aFL-GFP and G9aCat-NLS-GFP expression in mouse zygotes on 5mC and 5hmC. **a** Shown are the representative images of PN4/5 stage zygotes stained with antibodies against 5mC together with anti-ssDNA antibodies, or together with 5hmC antibodies. *m* Maternal pronucleus, *p* paternal pronucleus, *pb* polar body. *Scale bar* 50 µm. **b** Quantification of 5mC signals, normalized against ssDNA signals in both parental genomes of zygotes at PN4/5. Relative signal intensities in control groups are set to 1. Statistical significance was calculated using *t* test (***P < 0.001)

However, such strong increase in H3K9me2 signals in both pronuclei did not induce a major increase in (paternal) 5mC (Fig. 10). To examine the overall effect on 5mC/5hmC, we performed hairpin bisulphite sequencing of three repetitive elements: Line1 (L1Tf), intracisternal A-particle element (IAP) and major satellites (mSat) on late stage zygotes (i.e. after replication). Neither of these elements showed a significant increase in total methylation or hemimethylated sites when G9aCat-NLS-GFP injected group was compared with non-injected one (Additional file 8). We conclude that the increase in H3K9me2 alone does not directly control the genome-wide amount and replication-dependent persistence of DNA methylation. Our results are in line with a report by Liu et al., who also observed no visible changes in 5mC level (also visualized by immunostaining), despite the global increase in H3K9me2 on the paternal genome, caused by cycloheximide treatment of mouse zygotes [7].

Having shown that H3.1-GFPT11A mutant can be methylated by G9aCat-NLS-GFP in *E. coli* (see above), we next asked whether the decrease in H3K9me2, caused by the expression of H3T11A mutants, may be compensated by ectopic G9a overexpression in the mouse zygote. We co-injected mRNA encoding G9aCat-NLS-GFP with either H3.1-GFPT11A or H3.2-GFPT11A or H3.3-GFPT11A mRNAs, respectively. Indeed, the co-injection partially compensates for the H3K9me2 loss from maternal chromatin observed for H3.1-GFPT11A or H3.2-GFPT11A expressing groups alone, while the co-expression with H3.3T11A did not cause this effect and the G9A mediated H3K9me2 methylation was as high, as in zygotes, injected only with G9aCat-NLS-GFP alone (Additional file 9). The paternal chromatin, which is naturally depleted with H3K9me2, had an increased amount of this modification in all three mutated H3 variants (Additional file 9b). These results support the notion that the maintenance of H3K9me2 on H3.1 and H3.2 (and partially on H3.3) during replication is controlled by the H3T11 phosphorylation status.

## Discussion

**Fig. 11:**
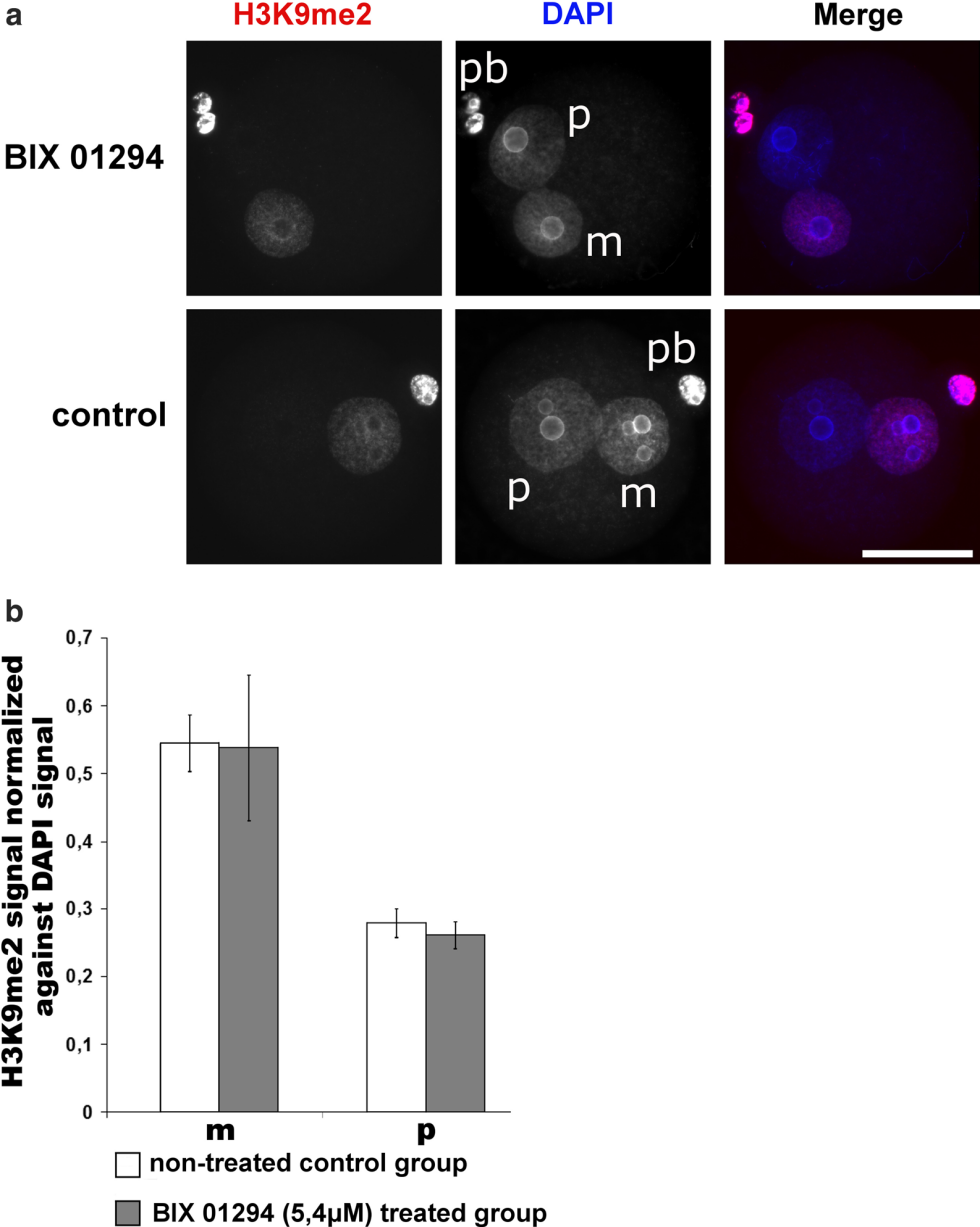
Influence of G9a-specific inhibitor BIX 01294 on H3K9me2 in mouse zygotes. Zygotes were incubated with the inhibitor (5,4 μM) starting from 2 h post-fertilization until 12 h and then fixed for immunostaining. **a** Shown are the representative images of PN4/5 stage zygotes stained with antibodies against H3K9me2. DNA is visualized by DAPI. *m* Maternal pronucleus, *p* paternal pronucleus, *pb* polar body. *Scale bar* 50 µm. **b** Quantification of H3K9me2 signals, normalized against DNA signals in both parental genomes of zygotes at PN4/5. Statistical significance was calculated using *t* test (***P < 0.001)

**Fig. 12:**
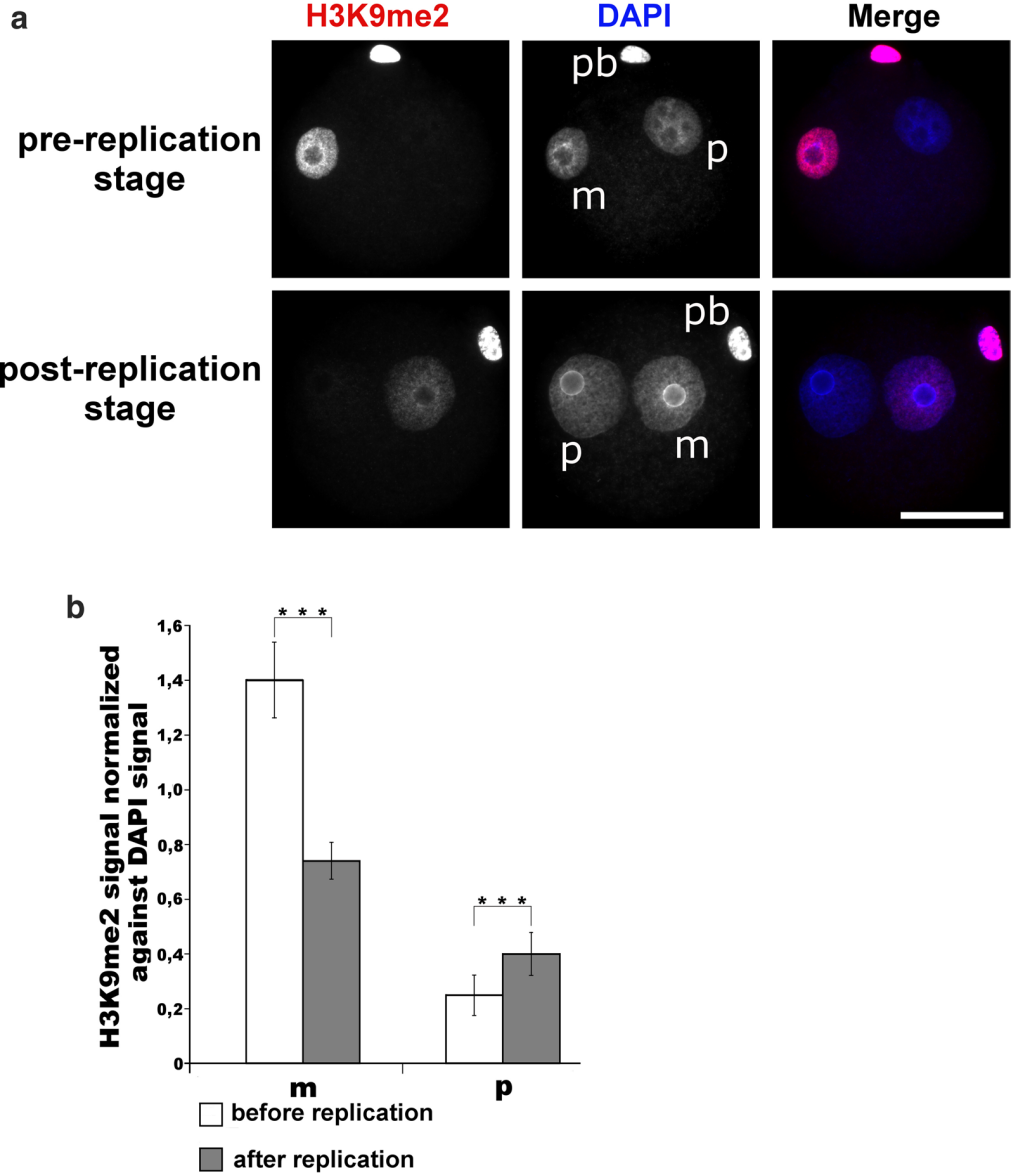
Relative abundance of H3K9me2 in mouse zygotes before and after replication. **a** Shown are the representative images of PN4/5 stage zygotes stained with antibodies against H3K9me2. DNA is visualized by DAPI. *m* Maternal pronucleus, *p* paternal pronucleus, *pb* polar body. *Scale bar* 50 µm. **b** Quantification of H3K9me2 signals, normalized against DNA signals in both parental genomes of zygotes at PN4/5. Statistical significance was calculated using *t* test (***P < 0.001)

**Fig. 13:**
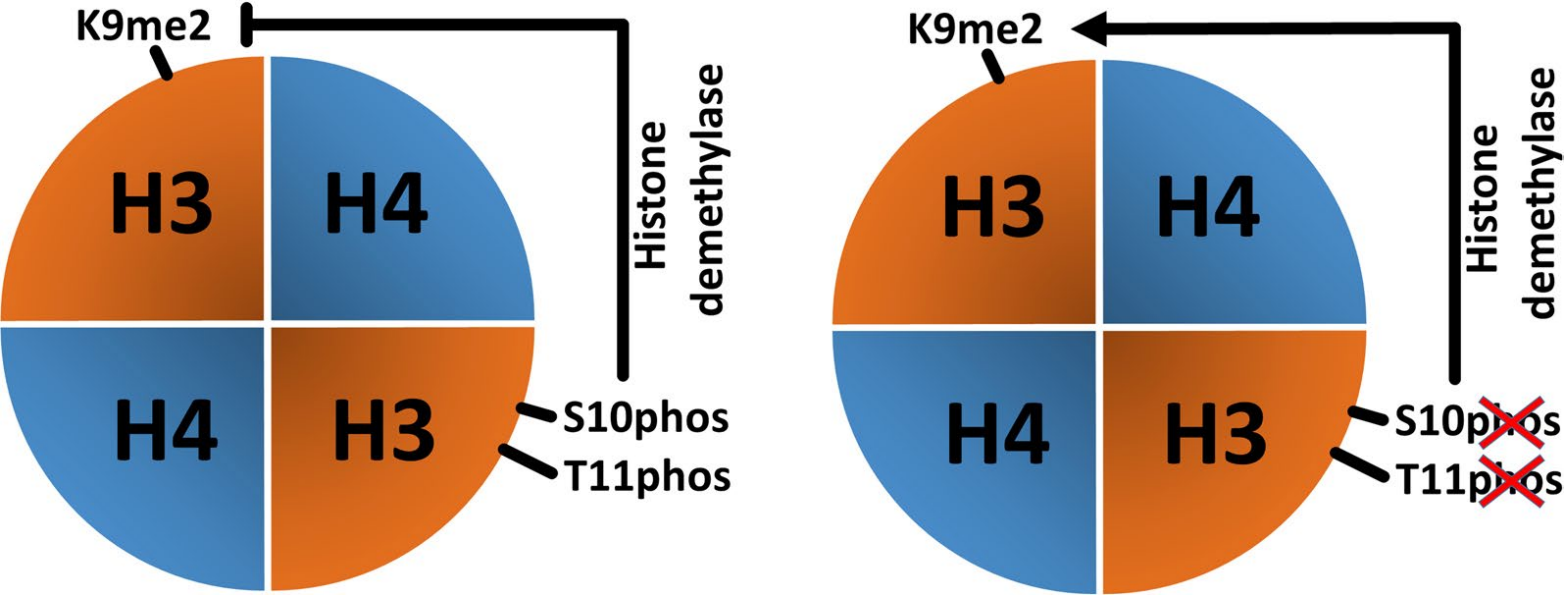
Schematic model of interaction of H3S10phos/H3T11phos with H3K9me2 within the nucleosome in mouse zygote. The presence of phosphorylation marks stabilizes H3K9me2, presumably by protecting from H3K9-specific histone demethylase

In our work, we investigated the possible dependency of the phosphorylation status at H3S10 and H3T11, the establishment and replication-dependent maintenance of H3K9me2 and the conversion of 5mC to 5hmC. Our findings add new facets to the role and crosstalk between histone modifications in epigenetic reprogramming of the zygote. In the zygote, H3K9me2 is a key epigenetic signal mainly inherited from the maternal chromosomes of the oocyte [2, 5, 6]. It remains an open question how the pronuclear asymmetry of H3K9me2 is maintained throughout the first cell cycle. De novo H3K9 dimethylation activities appear to be largely absent in the zygote [7]. Our findings support this assumption but also indicate some low-level de novo H3K9me2 activity on both paternal and maternal chromatin during late S- or G2-phase [7]. The nature of H3K9me2 modifying enzymes remains unclear. G9a, the main H3K9me2-specific methyltransferase, cannot be detected by immunofluorescence until four-cell stage (Additional file 6), and the inhibition of G9a by the specific inhibitor BIX 01294 does not cause any changes in the amount and distribution pattern of H3K9me2 in the zygote (Fig. 11). We also observe that the maternally inherited H3K9me2 signal is “halved” on nucleosomes after the first round of replication (Fig. 12a, b). We furthermore show that the H3K9me2 signal decreases when we overexpress either H3.1/2-GFPS10A or H3.1/2-GFPT11A mutated histones. Together these observations suggest that the phosphorylation controls the maintenance of H3K9me2. A “phospho-methylation” crosstalk has been proposed for H3K4me3. Here the demethylation by LSD1 is controlled by the absence of H3T6 phosphorylation [22]. A different crosstalk has been proposed for H3T11phos and H3K9me2 where phosphorylation triggers the demethylation in a cancer cell line. The authors suggested that the phosphorylation of H3T11 by PRK1 kinase accelerates the demethylation by JMJD2C histone demethylase [23]. While our findings also suggest a “phospho-methylation” crosstalk, they are obviously distinct from the cancer cell scenario. We observe (similar to the H3K4me3 example) that the absence of phosphorylation and not its presence triggers the reduction in (pre-existing) H3K9me2 in the zygote—predominantly in the maternal pronucleus. In the post-replicative chromatin, the newly assembled nucleosomes contain the “old” modified histones, probably as H3/H4 dimers, assembled with “new” unmodified H3/H4 dimers [24]. The phosphorylation of H3S10 or H3T11, which might occur on free H3 histones or on newly assembled H3/H4 dimers, appears to protect this “old” H3K9me2 probably by blocking a putative H3K9me2 demethylase (Fig. 13). In line with this scenario, we would like to note that the overexpression of non-methylatable H3.1K9R and H3.2K9R mutants strongly decreases T11 phosphorylation in maternal pronuclei (Additional file 10) and in consequence leads to a reduction (approx. 20%) in H3K9me2 as well as H3K9me3 (approx. 15%, see Additional file 11a, b).

We observe that dynamics and the variant-specific distribution of S10 and T11 phosphorylation are distinct and only partially overlapping. H3T11phos is preferentially linked to S-phase and the histone variants H3.1 and H3.2, while H3S10phos is present at G1 and G2 and found on all histone variants (Figs. 2, 3, respectively). Despite this differential dynamics, mutations in S10 and T11 phosphorylation sites cause very similar global effects on both H3K9me2 histone methylation and 5mC/5hmC DNA modifications. It remains to be clarified which regulatory proteins (histone demethylases or DNA modification controller) are influenced by S10 and T11 phosphorylation and how and where in the genome this occurs for the different histone H3 variants.

Some of our data suggest that the conversion of 5mC into 5hmC is directly linked to the histone phospho-methylation switch model discussed above. The overexpression of both H3.1/2-GFPS10A and H3.1/2-GFPT11A mutated histones indeed induces significant accumulations of 5hmC in the maternal pronucleus (Figs. 6a, 7a and Additional file 5). It is tempting to assume that the altered S10 and T11 phosphorylation influences changes in maternal H3K9me2 levels, which in turn influence the Tet3-mediated conversion of 5mC to 5hmC. However, one experiment argues against such a simple scenario. When overexpressing G9A in the zygote, we could massively increase the levels of H3K9 without comparably dramatic changes in the presence or the ratio of 5mC and 5hmC in maternal and paternal pronuclei (Fig. 10a, b). Hence, while the decrease in maternal H3K9me2 causes a strong change on maternal 5hmC amounts, the G9aCat-NLS-GFP-induced increase in H3K9me2 mainly in the paternal chromosome had no or little influence on 5hmC (Fig. 10a). This finding indicates that H3K9me2 (in combination with Stella) might not constitute the exclusive chromatin modification protecting chromatin against Tet-mediated oxidation.

In all our IF experiments, we observed a strong decrease in 5mC and an increase in 5hmC in zygotes overexpressing S10 and T11 mutants. When examining the DNA methylation patterns by hairpin bisulphite sequencing after the first DNA replication, we observe a massive increase in hemimethylated sites in zygotes overexpressing H3.1-GFPT11A mutants (Fig. 8). A lack of T11 (and probably also S10) phosphorylation apparently increases the presence of 5hmC, which in turn affects the maintenance of full methylation after DNA replication. This observation is in line with our previous findings, showing that 5hmC may interfere with DNA methylation maintenance [20, 21].

## Conclusions

Our findings provide novel insights into the crosstalk between DNA and histone modifications in the mammalian zygote. We find that serine 10 and threonine 11 phosphorylation of histone H3 shows a different dynamic and pronuclear appearance during the first cell cycle. Overexpression of variant-specific serine 10 and threonine 11 mutants reveals that the phosphorylation at both positions is important for the maintenance of the maternally inherited H3K9me2 as well as for the maintenance of symmetric CpG DNA methylation (5mC/5hmC). Mechanistically our data suggest that H3S10phos and H3T11phos help to stabilize H3K9me2 during the first cell cycle most likely by protecting maternal chromatin against a K9-specific demethylation activity.

## Methods

### Mouse superovulation and in vitro fertilization

For superovulation, females were injected with 6 IU pregnant mare serum gonadotropin (PMSG) and 6 IU human chorionic gonadotropin (hCG) with a time interval of around 48 h. Spermatozoa isolation, oocytes collection and IVF procedures were carried out as previously published protocol with small modifications [25]. Briefly, sperm was isolated from the cauda epididymis of male mice and capacitated in pre-gassed modified KSOM medium supplemented with 30 mg/ml BSA for 2 h. Mature oocytes were collected 15 h post-hCG injection of female mice. Cumulus–oocyte complexes were placed into a 400-μl drop of KSOM medium, mixed with capacitated sperm and incubated at 37 °C in a humidified atmosphere of 5% CO_2_ and 95% air.

### Plasmids preparation

The cDNAs of H3.1, H3.2 (gifts from Prof. Dr. Fugaku Aoki), H3.3 (purchased from ImaGenes GmbH) were cloned to pEGUP1 (a modified pET28b vector containing GFP coding sequence) by using NcoI and XhoI sites. The cDNA of G9a full length (gift from Prof. Dr. Y. Shinkai, Prof. Dr. M. Tachibana, Prof. Dr. M. Brand and Prof. Dr. M. R. Stallcup) was subcloned to pEGUP1. The same strategy was applied to G9aCat-GFP, HDAC1 (obtained from Addgene) and HDAC2 (gift from Prof. Dr. Richard M. Schultz). To create G9aCat-NLS-GFP expressing construct, G9aCat encoding DNA fragment was inserted to pEGUP1-NLS (pEGUP1 version with nuclear localization signal). For co-expression experiments in *E. coli*, G9aCat encoding fragment was cloned into pET15Am (a modified pET28b containing ampicillin resistance marker, p15 replication origin and no His-tag sequence). All positive constructs were verified by Sanger sequencing. All the primers used are listed in Additional file 12, also including the ones for mutagenesis, in vitro transcription as well as hairpin bisulphite sequencing.

### Site-specific mutagenesis

The generation of all histone H3 mutants (K9R, S10A, T11A) was done by overlapping PCR-based mutagenesis. Briefly, two PCRs, namely PCR1 and PCR2, were applied with two sets of primers in which reverse primer for PCR1 and forward primer for PCR2 are complementary and contain the desired mutation. After gel purification, the cleaned-up fragments from PCR1 and PCR2 were mixed as templates in overlapping PCR. The expected PCR products were then double-digested by NcoI and XhoI, followed by insertion into pEGUP1. The positive clones containing the corresponding mutations were selected and confirmed by Sanger sequencing.

### In vitro transcription of mRNAs

To prepare the DNA templates, highly purified DNA templates were extracted with phenol:chloroform, and capped mRNAs were generated by in vitro transcription using AmpliCap-Max T7 High Yield Message Maker Kit (CellScript, Inc.), followed by purification using RNA Clean & Concentrator™-25 Kit (Zymo research) according to the manufacturers’ instructions. Finally, transcribed mRNAs were eluted with nuclease-free water (Life technologies) and stored at −80 °C for later use.

### Expression of G9aCat and histone H3 variants in *E. coli*

For co-expression of G9aCat with histone H3.1-GFPWT, or H3.1-GFPT11A or H3.3-GFPWT, both constructs were transformed into Rosetta™ 2 Competent Cells. The transformed cells were grown in SOC medium with ampicillin (100 mg/mL) and kanamycin (25 mg/mL) until OD value reached 0.5–0.6 at 37 °C, followed by 0.5 mM isopropyl-b-d-thiogalactopyranoside (IPTG) induction for 24 h at 16 °C. Modified proteins of H3.1-GFPWT, H3.1-GFPT11A, H3.3-GFPWT were purified through Ni–NTA resin by taking advantage of the 6xHis tag. The protein expression was confirmed by Coomassie blue staining of SDS-PAGE gel. The presence of H3K9me2 or H3K9me3 on recombinant histones was verified by Western blotting using specific antibodies, which were also used for immunofluorescence.

The detailed information regarding the antibodies used is listed in Additional file 13.

### BIX 01294 treatment of zygotes

At 2 h post-fertilization, zygotes were collected and transferred into the 100 μl drop of KSOM medium containing BIX 01294 (5.4 μM). The zygotes were cultured to post-replication stages (PN4/5), followed by fixation for immunostaining.

### Microinjection of mRNAs into zygotes

At around 1hpf, just before microinjection, zygotes were collected, washed in M2 medium for several times and transferred into 100 μl drops of pre-gassed KSOM medium. Microinjection was performed under a Zeiss Axiovert 200 M inverted microscope (Zeiss) equipped with a FemtoJet microinjector and piezo-driven micromanipulators (Eppendorf). After a quick set-up, zygotes were placed into a 20-μl drop of M2 medium and cytoplasm injection was done with mRNAs encoding for H3.1/2/3-GFPWT/K9R/S10A/T11A, G9aFL-GFP, G9aCat-GFP, G9aCat-NLS-GFP or in combinations. Each microinjection experiment was replicated at least once, and groups of 10–30 zygotes were used for each experiment.

### Live monitoring chromatin incorporation of H3.1/2/3-GFPK9R and H3.1/2/3-GFPWT

After being microinjected with corresponding mRNAs, the zygotes were transferred into a KSOM drop of 10 µl on a glass bottom culture dish and placed into the chamber of Nikon BioStation live cell imaging System under the condition of 37 °C in a humidified atmosphere of 5% CO_2_ and 95% air. The pictures were captured every 15 min for 97 cycles with the exposure time 1/250 s.

### IF staining

The detailed protocol was described earlier elsewhere [10, 25]. After brief treatment in acidic Tyrode’s solution, zona-free zygotes and cleavage stage embryos were fixed by incubating in 3.7% paraformaldehyde solution in PBS for 30 min at RT, followed by permeabilization in 0.2% TritonX100/PBS at RT for 15 min. Then, the embryos were blocked at RT for 4 h or overnight at 4 °C in PBS, containing 0.1% TritonX100 and 3 mg/ml BSA. Later, the embryos were incubated with primary antibody overnight at 4 °C in blocking solution. Note that for 5mC, 5hmC, 5caC and ssDNA staining DNA was denatured: the fixed embryos were incubated in 4 M HCl for 15 min and then neutralized in 100 mM TrisCl pH 8.0 for 10 min at RT. For simultaneous 5hmC detection and PI DNA staining, the denaturation time was reduced to 6 min 30 s. After incubation with primary antibody, the embryos were washed 3–4 times in blocking solution and then incubated at RT for 2 h with fluorescently labelled secondary antibody. Finally, the embryos were washed and mounted on a glass slide in Vectashield mounting medium (Vector Laboratories). All antibodies used in this study are listed in Additional file 13.

### IF microscopy, quantification and statistical analysis

The mounted embryos were analysed on Zeiss Axiovert 200 M inverted microscope equipped with the fluorescence module and B/W digital camera for imaging. The IF images were captured, pseudocoloured and merged using AxioVision software (Zeiss). GIMP and Image J software were used together to complete quantification of the signals of z-stack computed images. For each group, at least 10 zygotes were analysed from at least two repeated experiments. Student’s *t* test was employed for statistical analysis.

### Hairpin bisulphite sequencing

Hairpin bisulphite high-throughput sequencing was performed as previously described with no modifications to the protocol [20].

## Additional files

**Additional file 1.** Localization of the ectopically expressed mutated histone H3 variants and G9a isoforms in zygotes. The direct visualization was enabled by the presence of GFP-tag fused to C-terminal part of the protein of interest. DNA was counterstained with DAPI.

**Additional file 2.** Influence of the overexpression of wild-type (WT) histone H3 variants fused to GFP on H3S10phos and H3T11phos at PN4/5. The figure provides three examples for each injection showing representative examples in which H3S10phos and H3T11phos patterns are either strongly changed (*top* example, few cases only), mildly changed (*middle*, less abundant) and unchanged (*bottom*, the majority of the injected zygotes) as compared to the uninjected control. DNA was visualized by DAPI. *Scale bar* 50 µm.

**Additional file 3.** Effects of H3.1-GFPT11A expression in mouse zygotes on H3K9me2 in direct comparison to H3.1-GFPWT expressing zygotes. Quantification of H3K9me2 signals, normalized against DNA signals in both parental genomes of zygotes at PN4/5. Relative signal intensities in control group are set to 1. Statistical significance was calculated using *t* test (****P* < 0.001).

**Additional file 4.** No interference of T11A mutation on H3.1-GFP with the ability of G9a to methylate histone H3 at K9 residue and with H3K9me2-specific antibody binding to its target. The WT or T11A mutated histone H3.1-GFP was expressed in *E. coli* either alone or together with G9aCat. The recombinant histone proteins were partially purified on Ni-NTA sepharose and applied to Western blot. *Lane 1* expression of H3.1-GFPWT alone; *lane 2* co-expression of H3.1-GFPWT and G9aCat; *lane 3* co-expression of H3.1-GFPT11A and G9aCat. *Upper panel* probing with anti-histone H3 antibody; *lower panel* probing with anti-H3K9me2 antibody.

**Additional file 5.** The influence of H3.1/2/3-GFPS10A or H3.1/2/3-GFPT11A expression on 5hmC in PN4/5 zygotes. The mean values (MV) of 5hmC signals in paternal (MVpat) or maternal (MVmat) pronuclei were calculated as integral signal density (ID) to area ratio (MVpat = IDpat/Area; MVmat = IDmat/Area). Plotted are MVpat to MVmat ratios. Statistical significance was calculated using *t* test (****P* < 0.001).

**Additional file 6.** Localization of the endogenously expressed G9a histone methyltransferase in pre-implantation embryos from zygote to blastocyst stages. Embryos at different stages were fixed and immunostained with antibodies against G9a. DNA is visualized by DAPI. *Scale bar* 50 µm.

**Additional file 7.** G9aFL-GFP and G9aCat-NLS-GFP expression in mouse zygotes has no effect on H3K9me3. Shown are the representative images of PN4/5 stage zygotes stained with antibodies against H3K9me3. DNA is visualized by DAPI. *m* Maternal pronucleus, *p* paternal pronucleus, *pb* polar body. *Scale bar* 50 µm.

**Additional file 8.** Hairpin bisulphite sequencing profiles of Line1, IAP and mSat repetitive elements in PN4/5 zygotes expressing G9aCat-NLS-GFP or in non-injected control group. *Bars* represent the sum of the DNA methylation status of all CpG dyads. The *map* next to the *bar* represents the distribution of methylated sites. Each *column* shows individual neighbouring CpG dyads, and each *line* represents one sequence read. The reads in the *map* are sorted according to their methylation status.

**Additional file 9.** Effects of H3.1/2/3-GFPT11A co-expression with G9amCat-NLS-GFP in mouse zygotes on H3K9me2. **a** Shown are the representative images of PN4/5 stage zygotes stained with antibodies against H3K9me2. DNA is visualized by DAPI. *m* Maternal pronucleus, *p* paternal pronucleus, *pb* polar body. *Scale bar* 50 µm. **b** Quantification of H3K9me2 signals, normalized against DNA signals in both parental genomes of zygotes at PN4/5. Relative signal intensities in control groups are set to 1. Statistical significance was calculated using *t* test (****P* < 0.001).

**Additional file 10.** Effects of H3.1/2/3-GFPK9R expression in mouse zygotes on H3T11phos. Shown are the representative images of PN4/5 stage zygotes stained with antibodies against H3T11phos. DNA is visualized by DAPI. *m* Maternal pronucleus, *p* paternal pronucleus, *pb* polar body. *Scale bar* 50 µm.

**Additional file 11.** Effects of H3.1/2/3-GFPK9R or wild-type H3.1/2/3-GFP expression in mouse zygotes on **a** H3K9me2 and **b** H3K9me3. H3K9me2 and H3K9me3 signals were quantified and normalized against DNA signals in either both parental genomes (for H3K9me2) or maternal genomes only (for H3K9me3, due to virtually absent signals in paternal pronuclei) of zygotes at PN4/5. Relative signal intensities in control (non-injected) groups are set to 1. Statistical significance was calculated using *t* test (****P* < 0.001; ***P* < 0.01; **P* < 0.05).

**Additional file 12.** The list and sequences of primers used in this study.

**Additional file 13.** The list and detailed information about the antibodies used in this study.

## Abbreviations

H3S10: histone H3 serine 10
H3T11: histone H3 threonine 11
H3S10phos: histone H3 serine 10 phosphorylation
H3T11phos: histone H3 threonine 11 phosphorylation
H3K9me2: histone H3 lysine 9 dimethylation
H3K9me3: histone H3 lysine 9 trimethylation
H3K4me3: histone H3 lysine 4 trimethylation
5mC: 5-methylcytosine
5hmC: 5-hydroxymethylcytosine
WT: wild-type
IF: immunofluorescence
G9aCat: catalytical domain of G9a histone methyltransferase
G9aFL: full-length G9a histone methyltransferase
IAP: intracisternal A-particle element
mSat: major satellites
NLS: nuclear localization signal
PMSG: pregnant mare serum gonadotropin
hCG: human chorionic gonadotropin
