# Supplementary figures and images for: Histone and DNA methylation control by H3 serine 10/threonine 11 phosphorylation in the mouse zygote
Source: Epigenetics Chromatin. 2017 Feb 14;10:5. doi: 10.1186/s13072-017-0112-x (PMC5307733; doi:10.1186/s13072-017-0112-x)

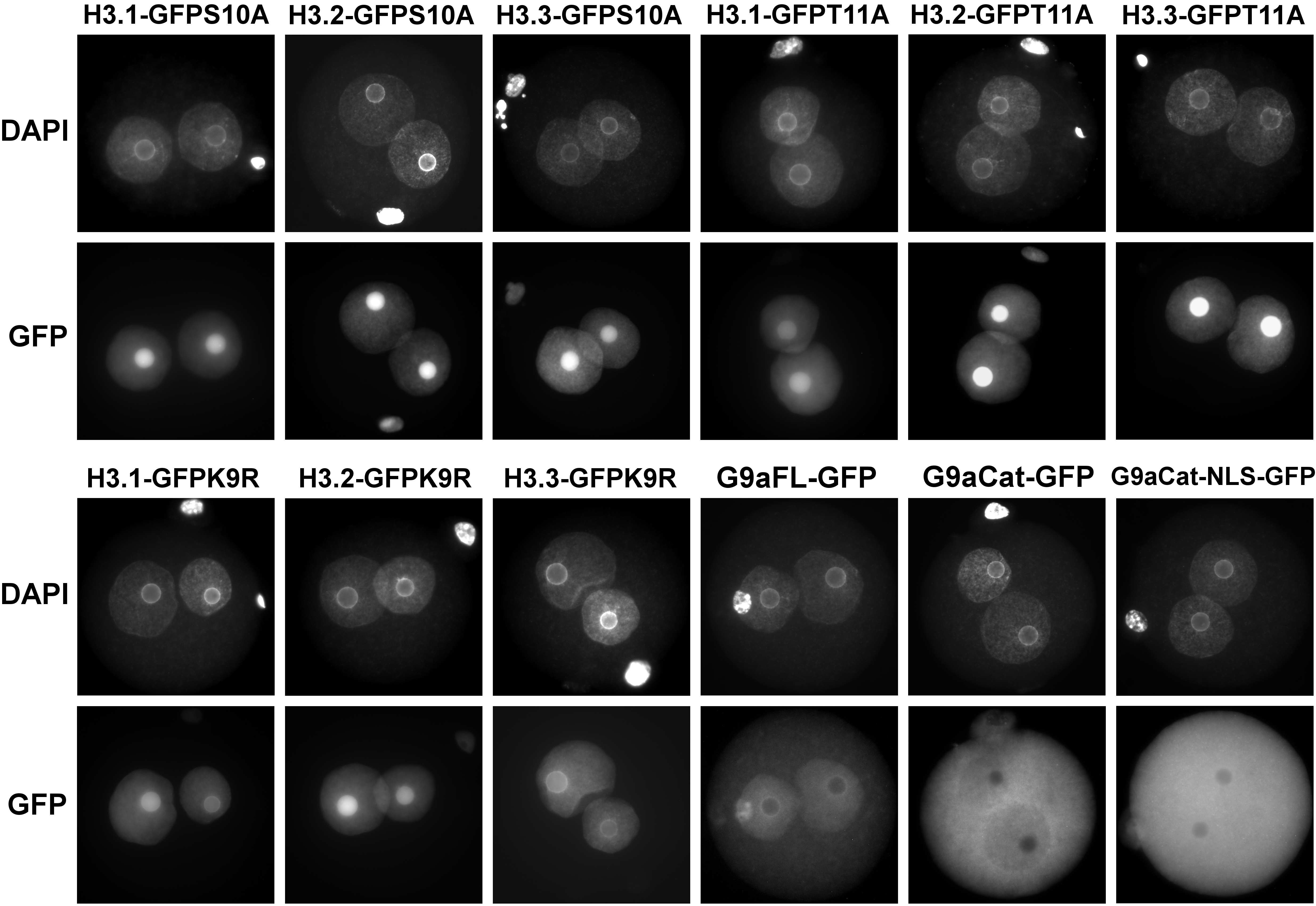

Supplement: Supplementary file 1 — Additional file 1. Localization of the ectopically expressed mutated histone H3 variants and G9a isoforms in zygotes. The direct visualization was enabled by the presence of GFP-tag fused to C-terminal part of the protein of interest. DNA was counterstained with DAPI. [file 13072_2017_112_MOESM1_ESM.jpg]

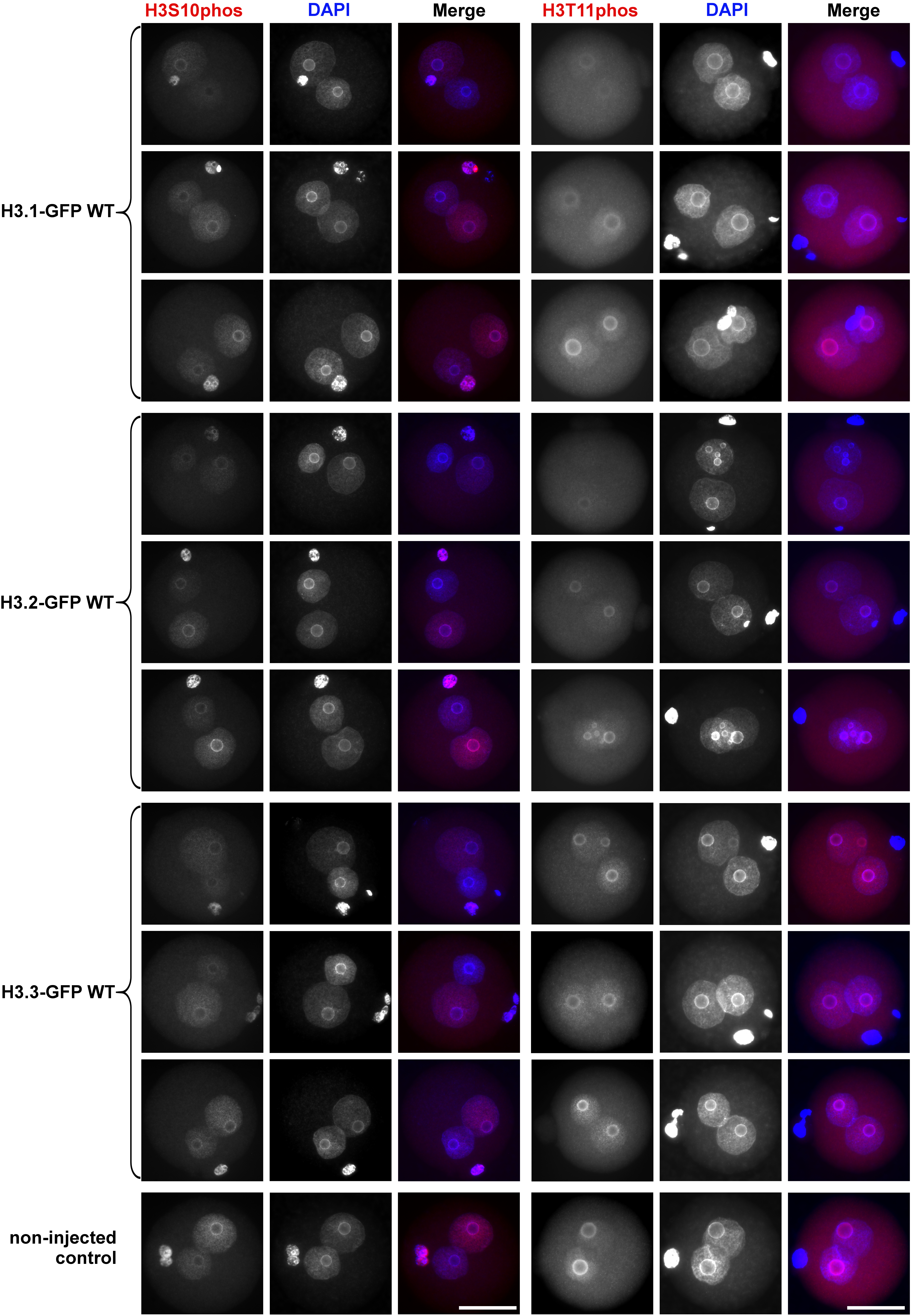

Supplement: Supplementary file 2 — Additional file 2. Influence of the overexpression of wild-type (WT) histone H3 variants fused to GFP on H3S10phos and H3T11phos at PN4/5. The figure provides three examples for each injection showing representative examples in which H3S10phos and H3T11phos patterns are either strongly changed (top example, few cases only), mildly changed (middle, less abundant) and unchanged (bottom, the majority of the injected zygotes) as compared to the uninjected control. DNA was visualized by DAPI. Scale bar 50 µm. [file 13072_2017_112_MOESM2_ESM.jpg]

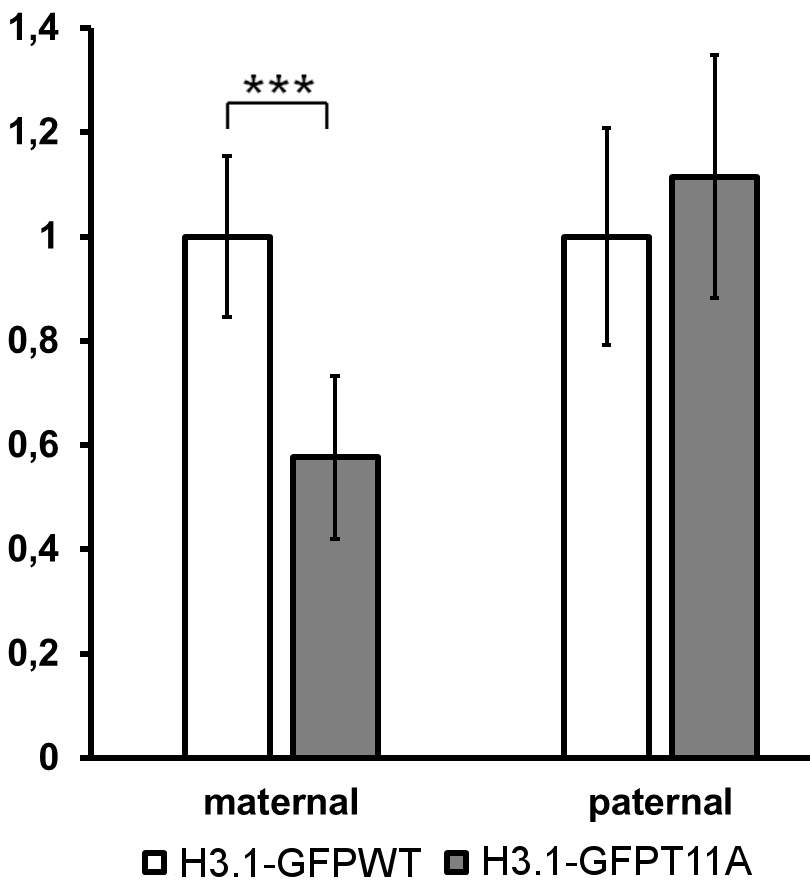

Supplement: Supplementary file 3 — Additional file 3. Effects of H3.1-GFPT11A expression in mouse zygotes on H3K9me2 in direct comparison to H3.1-GFPWT expressing zygotes. Quantification of H3K9me2 signals, normalized against DNA signals in both parental genomes of zygotes at PN4/5. Relative signal intensities in control group are set to 1. Statistical significance was calculated using t test (***P < 0.001). [file 13072_2017_112_MOESM3_ESM.jpg]

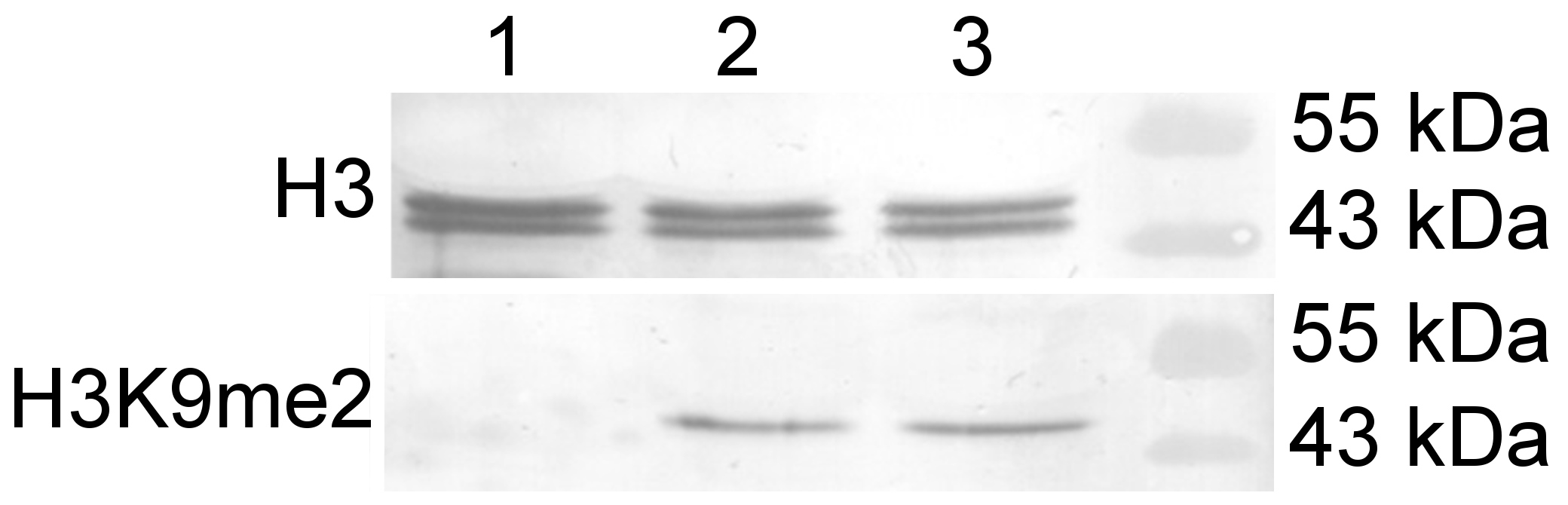

Supplement: Supplementary file 4 — Additional file 4. No interference of T11A mutation on H3.1-GFP with the ability of G9a to methylate histone H3 at K9 residue and with H3K9me2-specific antibody binding to its target. The WT or T11A mutated histone H3.1-GFP was expressed in E. coli either alone or together with G9aCat. The recombinant histone proteins were partially purified on Ni-NTA sepharose and applied to Western blot. Lane 1 expression of H3.1-GFPWT alone; lane 2 co-expression of H3.1-GFPWT and G9aCat; lane 3 co-expression of H3.1-GFPT11A and G9aCat. Upper panel probing with anti-histone H3 antibody; lower panel probing with anti-H3K9me2 antibody. [file 13072_2017_112_MOESM4_ESM.jpg]

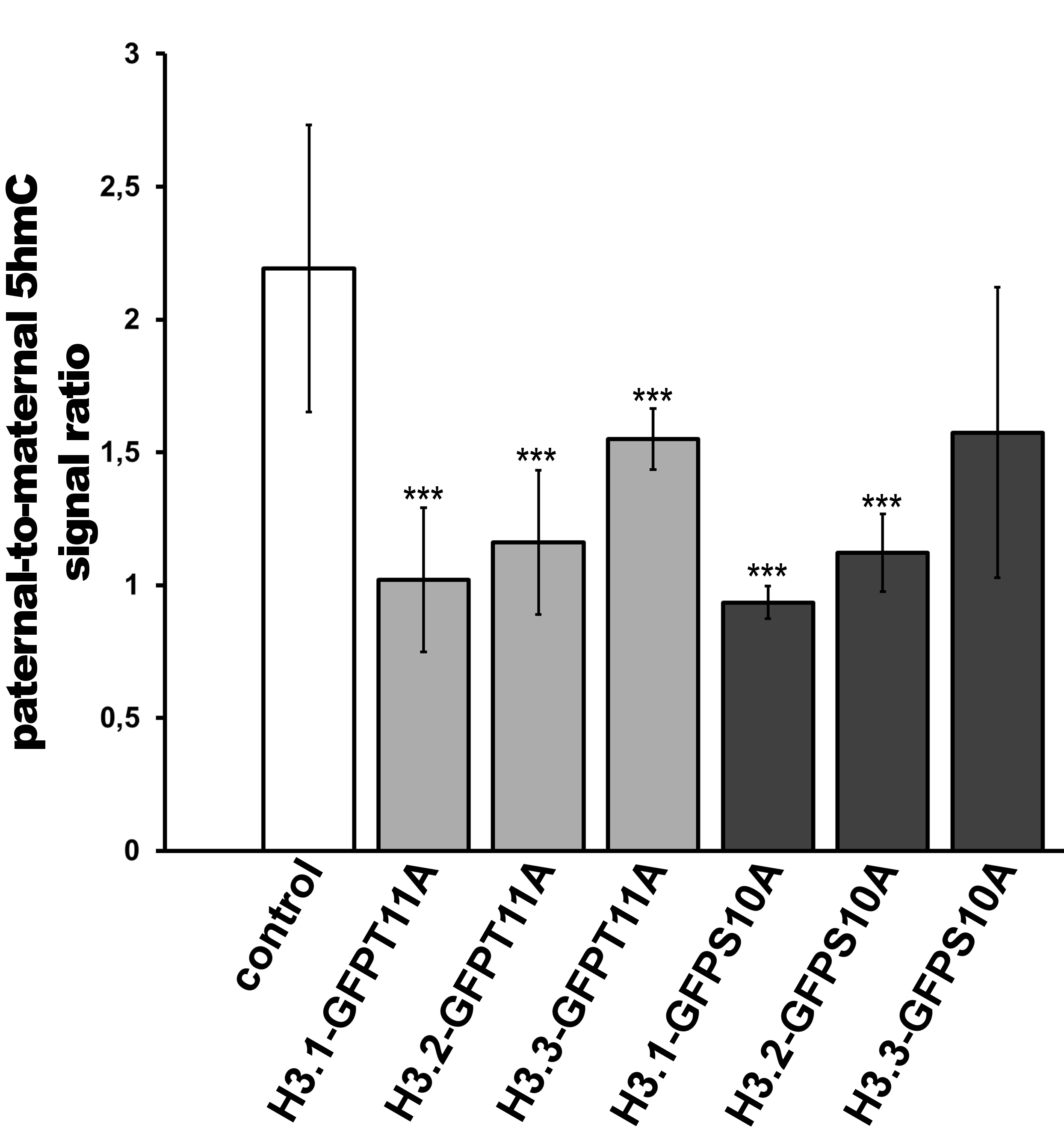

Supplement: Supplementary file 5 — Additional file 5. The influence of H3.1/2/3-GFPS10A or H3.1/2/3-GFPT11A expression on 5hmC in PN4/5 zygotes. The mean values (MV) of 5hmC signals in paternal (MVpat) or maternal (MVmat) pronuclei were calculated as integral signal density (ID) to area ratio (MVpat = IDpat/Area; MVmat = IDmat/Area). Plotted are MVpat to MVmat ratios. Statistical significance was calculated using t test (***P < 0.001). [file 13072_2017_112_MOESM5_ESM.jpg]

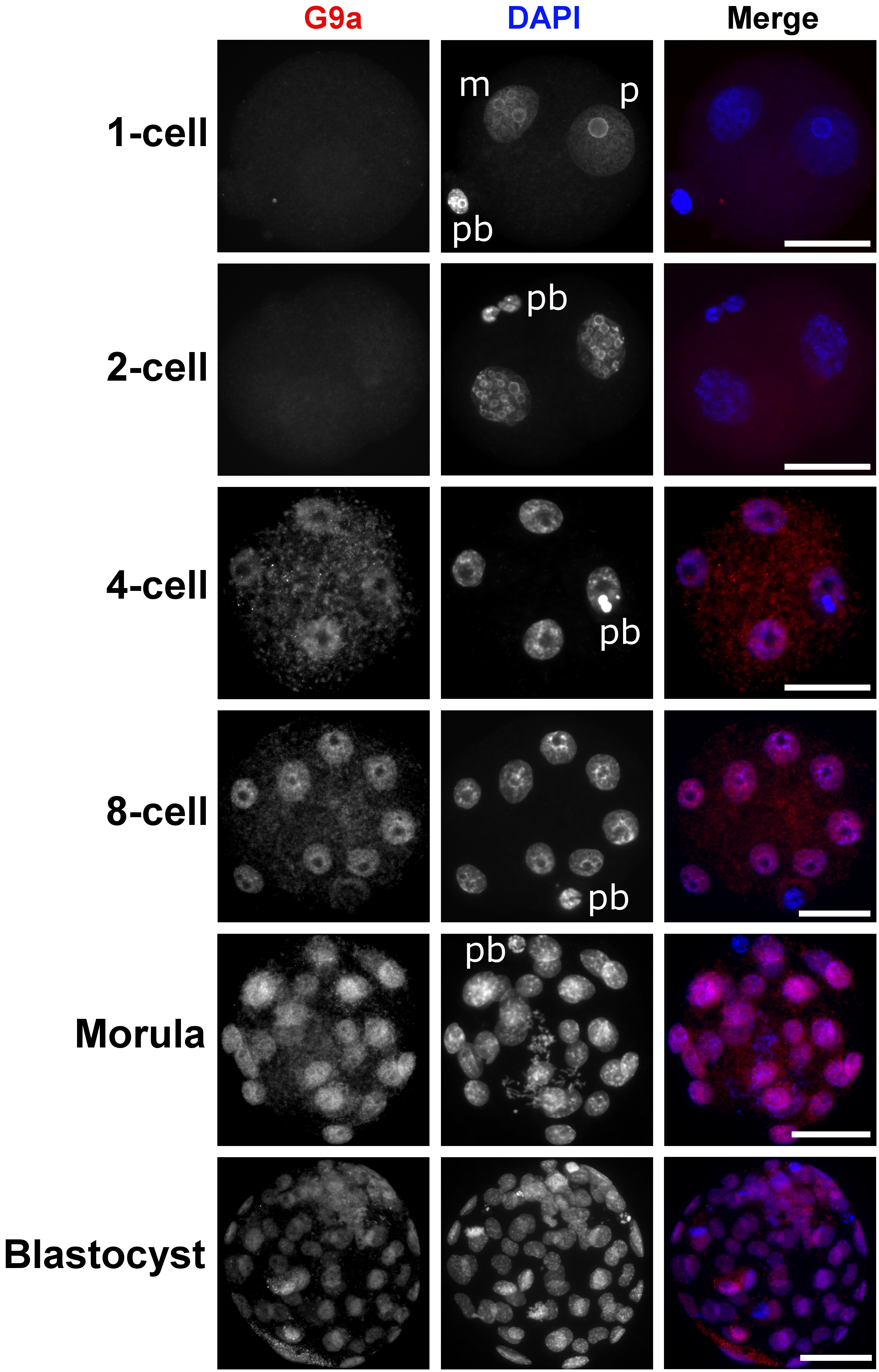

Supplement: Supplementary file 6 — Additional file 6. Localization of the endogenously expressed G9a histone methyltransferase in pre-implantation embryos from zygote to blastocyst stages. Embryos at different stages were fixed and immunostained with antibodies against G9a. DNA is visualized by DAPI. Scale bar 50 µm. [file 13072_2017_112_MOESM6_ESM.jpg]

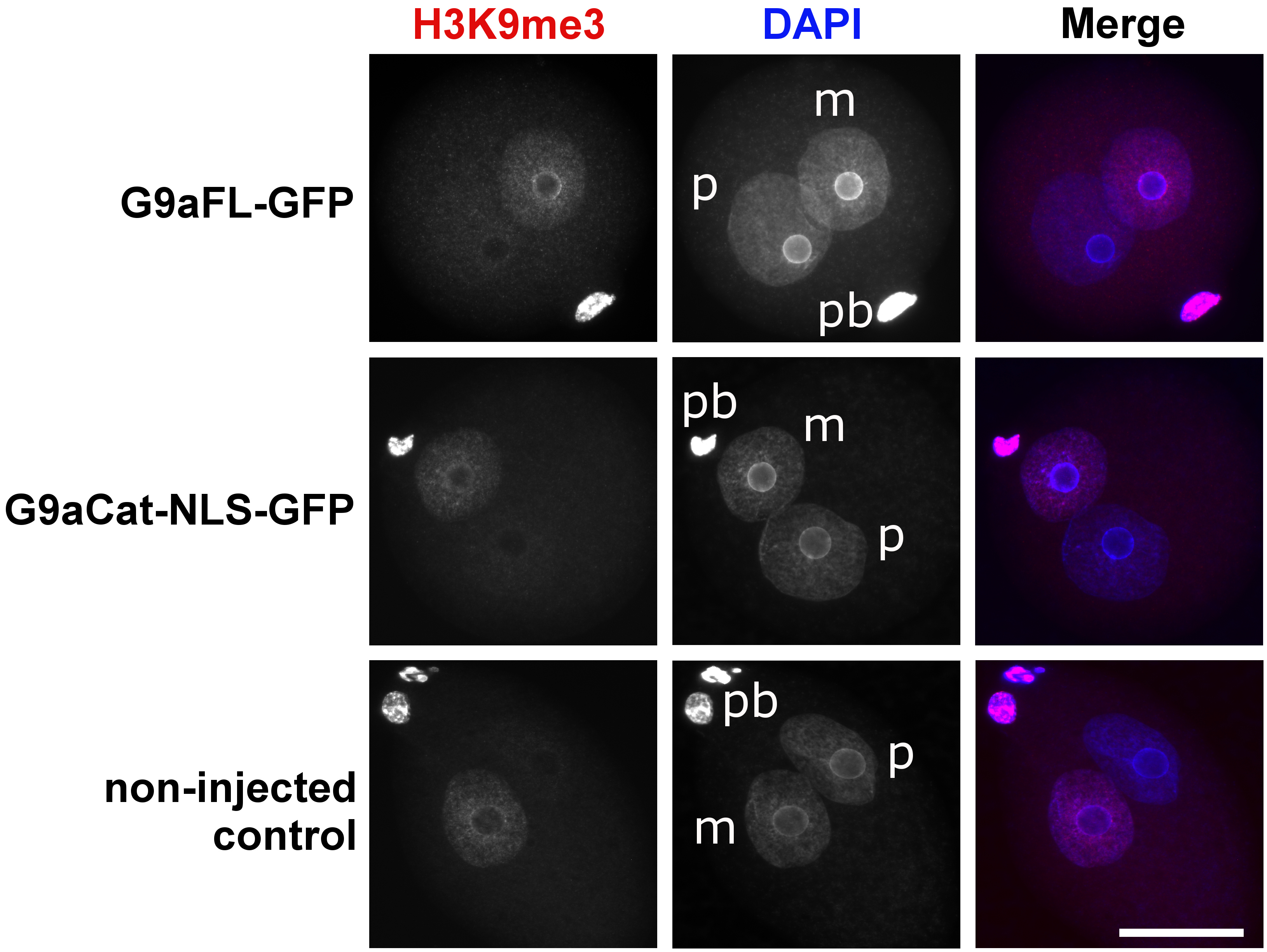

Supplement: Supplementary file 7 — Additional file 7. G9aFL-GFP and G9aCat-NLS-GFP expression in mouse zygotes has no effect on H3K9me3. Shown are the representative images of PN4/5 stage zygotes stained with antibodies against H3K9me3. DNA is visualized by DAPI. m Maternal pronucleus, p paternal pronucleus, pb polar body. Scale bar 50 µm. [file 13072_2017_112_MOESM7_ESM.jpg]

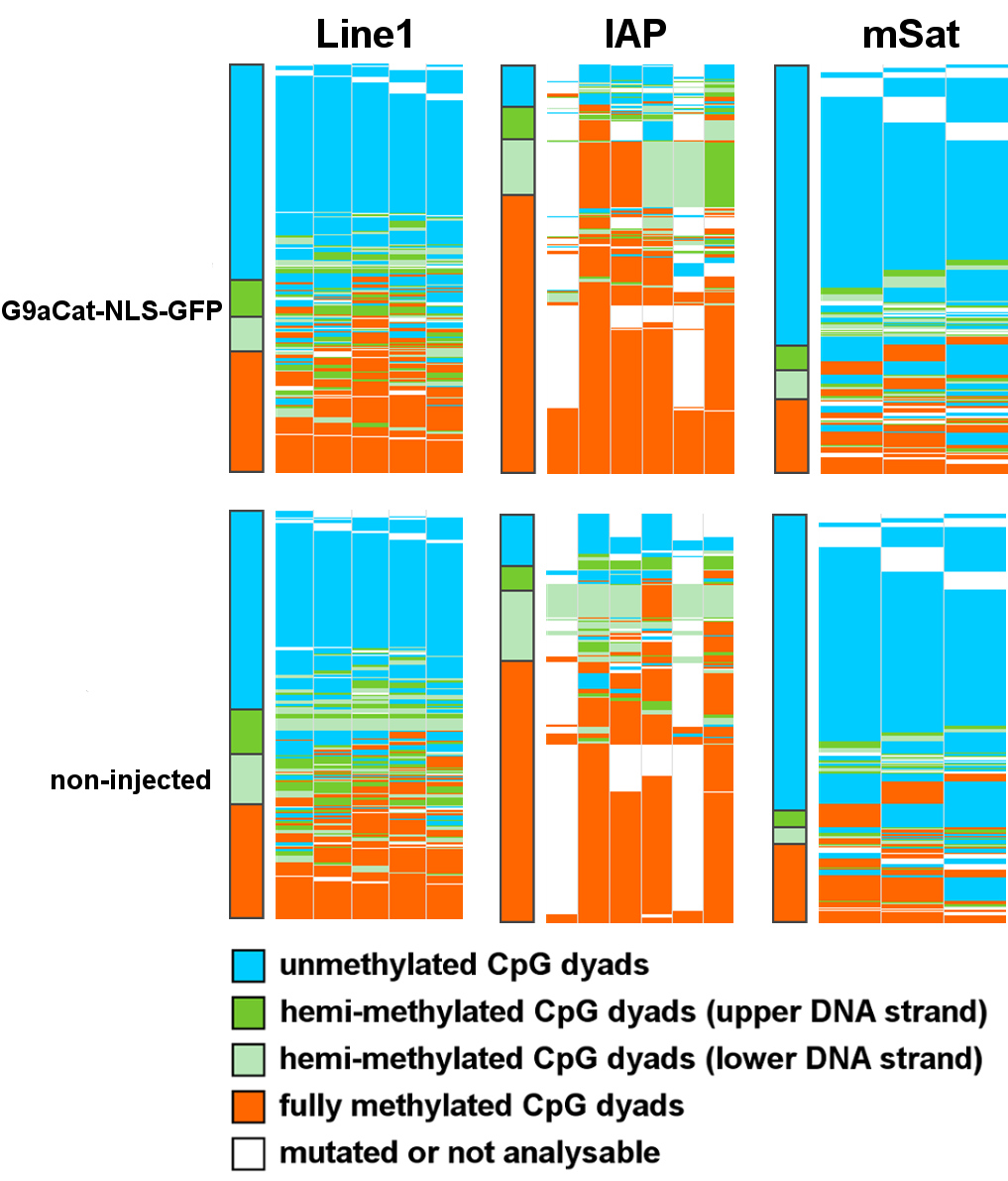

Supplement: Supplementary file 8 — Additional file 8. Hairpin bisulphite sequencing profiles of Line1, IAP and mSat repetitive elements in PN4/5 zygotes expressing G9aCat-NLS-GFP or in non-injected control group. Bars represent the sum of the DNA methylation status of all CpG dyads. The map next to the bar represents the distribution of methylated sites. Each column shows individual neighbouring CpG dyads, and each line represents one sequence read. The reads in the map are sorted according to their methylation status. [file 13072_2017_112_MOESM8_ESM.jpg]

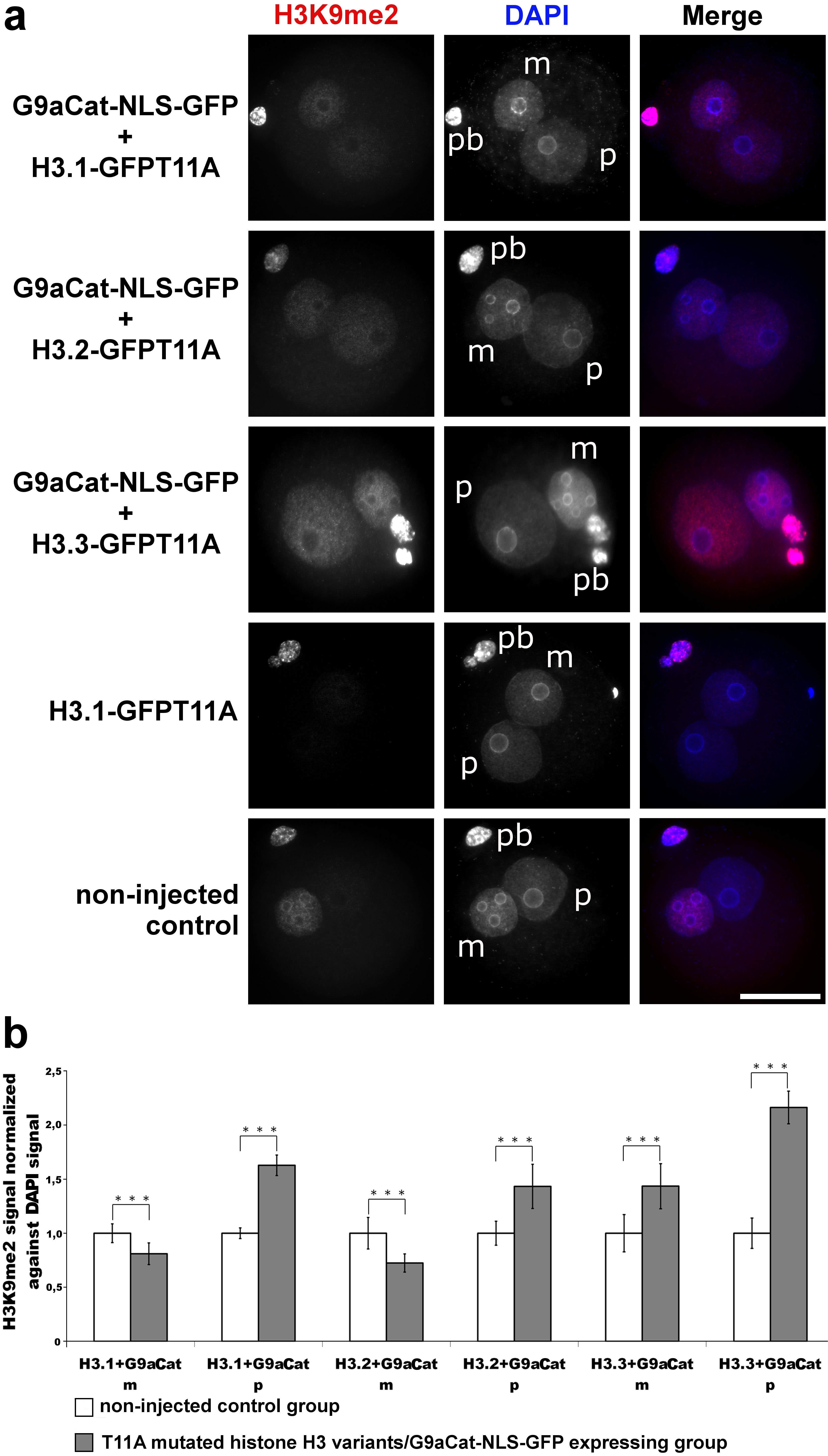

Supplement: Supplementary file 9 — Additional file 9. Effects of H3.1/2/3-GFPT11A co-expression with G9amCat-NLS-GFP in mouse zygotes on H3K9me2. a Shown are the representative images of PN4/5 stage zygotes stained with antibodies against H3K9me2. DNA is visualized by DAPI. m Maternal pronucleus, p paternal pronucleus, pb polar body. Scale bar 50 µm. b Quantification of H3K9me2 signals, normalized against DNA signals in both parental genomes of zygotes at PN4/5. Relative signal intensities in control groups are set to 1. Statistical significance was calculated using t test (***P < 0.001). [file 13072_2017_112_MOESM9_ESM.jpg]

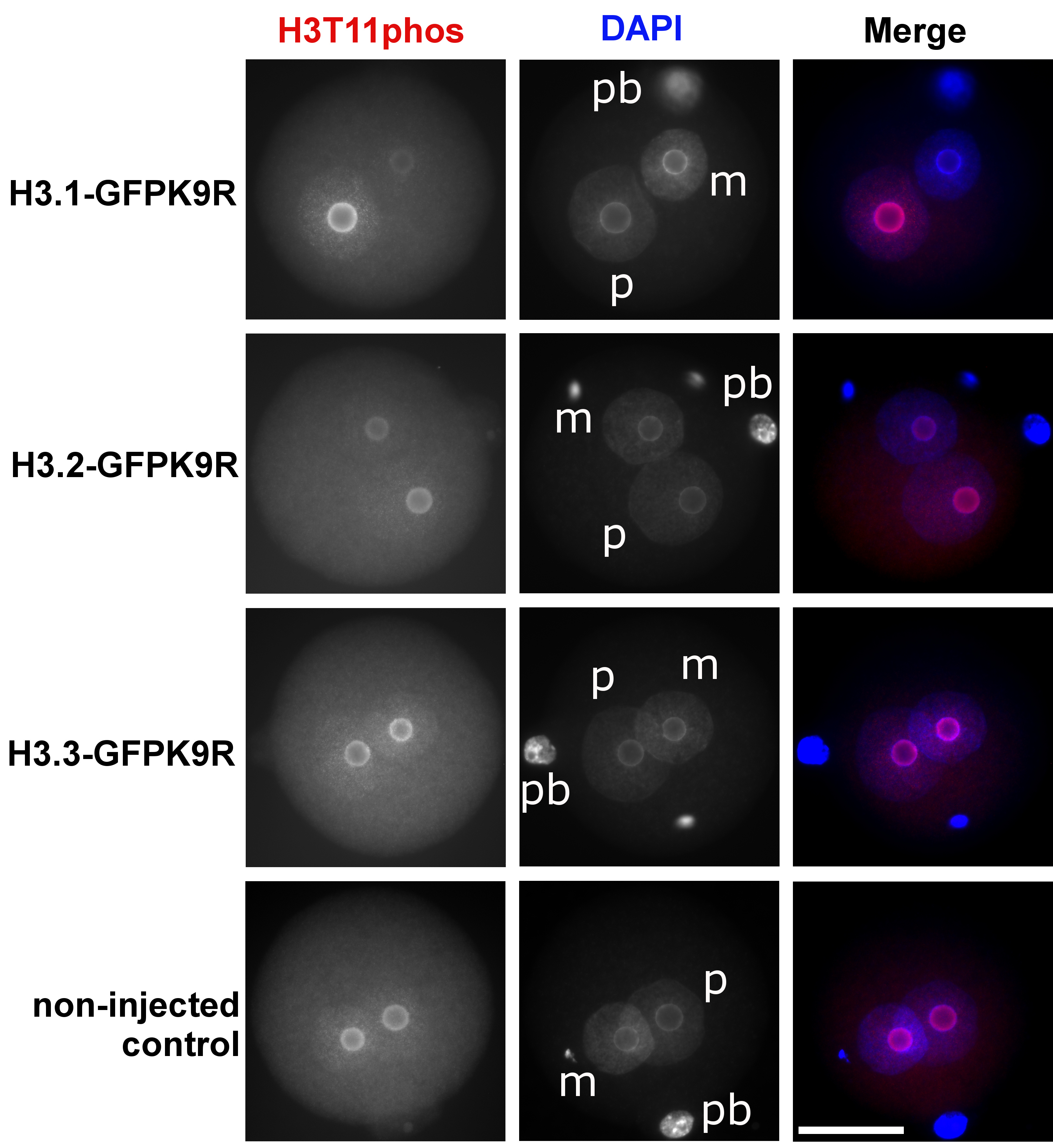

Supplement: Supplementary file 10 — Additional file 10. Effects of H3.1/2/3-GFPK9R expression in mouse zygotes on H3T11phos. Shown are the representative images of PN4/5 stage zygotes stained with antibodies against H3T11phos. DNA is visualized by DAPI. m Maternal pronucleus, p paternal pronucleus, pb polar body. Scale bar 50 µm. [file 13072_2017_112_MOESM10_ESM.jpg]

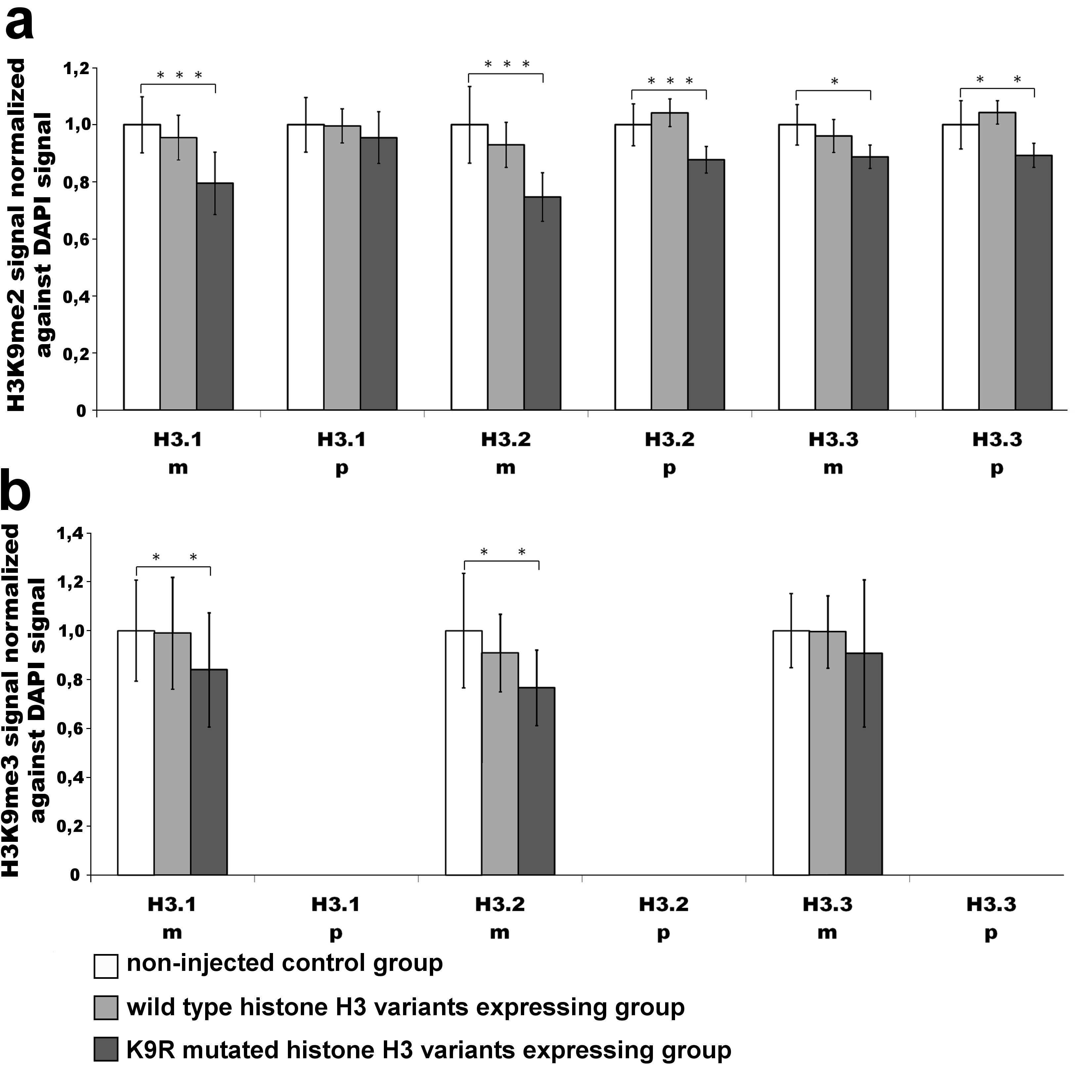

Supplement: Supplementary file 11 — Additional file 11. Effects of H3.1/2/3-GFPK9R or wild-type H3.1/2/3-GFP expression in mouse zygotes on a H3K9me2 and b H3K9me3. H3K9me2 and H3K9me3 signals were quantified and normalized against DNA signals in either both parental genomes (for H3K9me2) or maternal genomes only (for H3K9me3, due to virtually absent signals in paternal pronuclei) of zygotes at PN4/5. Relative signal intensities in control (non-injected) groups are set to 1. Statistical significance was calculated using t test (***P < 0.001; **P < 0.01; *P < 0.05). [file 13072_2017_112_MOESM11_ESM.jpg]
