# Supplementary material for: Histone and DNA methylation control by H3 serine 10/threonine 11 phosphorylation in the mouse zygote
Source: Epigenetics Chromatin. 2017 Feb 14;10:5. doi: 10.1186/s13072-017-0112-x (PMC5307733; doi:10.1186/s13072-017-0112-x)
Supplement: Supplementary file 12 — Additional file 12. The list and sequences of primers used in this study. [file 13072_2017_112_MOESM12_ESM.doc]

**Primer sequences**

| Name | Sequence (5’ to 3’) | Application |
| --- | --- | --- |
| H3.1-GFPWild type FWD | CATGCCATGGCTCGTACTAAGCAGACCGC | Cloning |
| H3.1-GFPWild type REV | CCGCTCGAGGCCGCCAGCCCTCTCCCCGCGGATGCGGC | Cloning |
| H3.2-GFPWild type FWD | CATGCCATGGCCCGTACGAAGCAGACTGC | Cloning |
| H3.2-GFPWild type REV | CCGCTCGAGGCCGCCAGCGCGCTCCCCACGGATGCGG | Cloning |
| H3.3-GFPWild type FWD | CATGCCATGGCTCGTACAAAGCAGACTGC | Cloning |
| H3.3-GFPWild type REV | CCGCTCGAGGCCGCCAGCACGTTCTCCGCGTATGCGGC | Cloning |
| MutGeneralPCR1 FWD | CCTGCCACCATACCCACGCCGAAAC | Mutagenesis |
| H3.1-GFPK9RPCR1 REV | GCCTTGCCGCCGGTAGAGCGGCGAGCGGTCTGCTTAGT | Mutagenesis |
| H3.1-GFPK9RPCR2 FWD | ACTAAGCAGACCGCTCGCCGCTCTACCGGCGGCAAGGC | Mutagenesis |
| MutGeneralPCR2 REV | CGTAGGTCAGGGTGGTCACGAGGGT | Mutagenesis |
| H3.2-GFPK9RPCR1 REV | GCCTTGCCGCCAGTGGAGCGGCGAGCGGTCTGCTTCG | Mutagenesis |
| H3.2-GFPK9RPCR2 FWD | CGAAGCAGACCGCTCGCCGCTCCACTGGCGGCAAGGC | Mutagenesis |
| H3.3-GFPK9RPCR1 REV | GTGCTTTACCACCGGTGGAGCGGCGGGCAGTCTGCTTTGT | Mutagenesis |
| H3.3-GFPK9RPCR2 FWD | ACAAAGCAGACTGCCCGCCGCTCCACCGGTGGTAAAGCAC | Mutagenesis |
| H3.1-GFPS10APCR1 REV | CGGGGCCTTGCCGCCGGTGGCCTTGCGAGCGGTCTGCT | Mutagenesis |
| H3.1-GFPS10APCR2 FWD | AGCAGACCGCTCGCAAGGCCACCGGCGGCAAGGCCCCG | Mutagenesis |
| H3.2-GFPS10APCR1 REV | CGGGGCCTTGCCGCCAGTGGCCTTGCGAGCGGTCTGCT | Mutagenesis |
| H3.2-GFPS10APCR2 FWD | AGCAGACCGCTCGCAAGGCCACTGGCGGCAAGGCCCCG | Mutagenesis |
| H3.3-GFPS10APCR1 REV | CTGGGTGCTTTACCACCGGTGGCTTTGCGGGCAGTCTGC | Mutagenesis |
| H3.3-GFPS10APCR2 FWD | GCAGACTGCCCGCAAAGCCACCGGTGGTAAAGCACCCAG | Mutagenesis |
| H3.1-GFPT11APCR1 REV | CGCGGGGCCTTGCCGCCGGCAGACTTGCGAGCGGTCTGC | Mutagenesis |
| H3.1-GFPT11APCR2 FWD | GCAGACCGCTCGCAAGTCTGCCGGCGGCAAGGCCCCGCG | Mutagenesis |
| H3.2-GFPT11APCR1 REV | CGCGGGGCCTTGCCGCCGGCGGACTTGCGAGCGGTCTGC | Mutagenesis |
| H3.2-GFPT11APCR2 FWD | GCAGACCGCTCGCAAGTCCGCCGGCGGCAAGGCCCCGCG | Mutagenesis |
| H3.3-GFPT11APCR1 REV | CTGGGTGCTTTACCACCGGCGGATTTGCGGGCAGTCTGC | Mutagenesis |
| H3.3-GFPT11APCR2 FWD | GCAGACTGCCCGCAAATCCGCCGGTGGTAAAGCACCCAG | Mutagenesis |
| G9aFL-GFP FWD | CGGGGTCTGCCGAGAGGGAGGGGGCTGATGCGGGCCCG | Cloning |
| G9aFL-GFP REV | CCGCTCGAGGGTGTTGATGGGGGGCAGGGAGCTGAG | Cloning |
| G9aCat FWD | GTCTTCTGTCCCCACTGTGGAG | Cloning |
| G9aCat REV | CCGCTCGAGTTAGGTGTTGATGGGGGGCAGGGAGCTGAG | Cloning |
| G9aCat-GFP FWD | GTCTTCTGTCCCCACTGTGGAG | Cloning |
| G9aCat-GFP REV | CCGCTCGAGGGTGTTGATGGGGGGCAGGGAGCTGAG | Cloning |
| G9aCat-NLS-GFP FWD | GTCTTCTGTCCCCACTGTGGAG | Cloning |
| G9aCat-NLS-GFP REV | TGGCCGACGTCGACGGTGTTGATGGGGGGCAGGGAGCTGAG | Cloning |
| LINE1 FWD | TGGTAGTTTTTAGGTGGTATAGAT | Sequencing |
| LINE1 REV | TCAAACACTATATTACTTTAACAATTCCCA | Sequencing |
| IAP FWD | TTTTTTTTTTAGGAGAGTTATATTT | Sequencing |
| IAP REV | ATCACTCCCTAATTAACTACAAC | Sequencing |
| mSAT FWD | GGAAAATTTAGAAATGTTTAATGTAG | Sequencing |
| mSAT REV | AACAAAAAAACTAAAAATCATAAAAA | Sequencing |
| T7 promoter | TAATACGACTCACTATAGGG | Sequencing |
| T7 upstream | TGCGTCCGGCGTAGAGGATCG | mRNA prep |
| T7 terminator | GCTAGTTATTGCTCAGCGG | mRNA prep |
| eGFP1 REV | GACACGCTGAACTTGTGGC | Sequencing |
| LINE1-Hairpin linker | Tggtagtttttaggtggtatagattctcacttaagcagactaaattcctaagttccttggagtcccgggaccaagatg  gcgaccgctgctgctgtggcttaggcgccccccagccgggcgggcacctgtcctccggtCCGGaGnGRC  CATnnnnnnnnATGGGRCCtccngaccggaggacaggtgcccgcccggctggggaggcggccT  aagccAcagcagcagcggtcgccatcttggtcccgggactccaaggaacttaggaatttagtctgcttaagtgag  agtctgtaccacctgggaattgttaaagtaatatagtgtttga | Sequencing |
| IAP-Hairpin linker | Tcccttttttaggagagttatatttcgccttagacgtgtcactccctgattggctgcagcccatcggccgagttgacgt  caAgttgacgtcacggggaaggcagagcacatggagtagagaaccacctcggcatatgcgcagattatttgttta  ccacTTAgggRTTatNNNNNNNNatgggRTTtaagtggtaaacaaataatctgcgcatatGccgag  ggtggttctctactccatgtgctctgccttccgtgacgtcaactcggccgatgggctgcagccaatcagggagtgac | Sequencing |
| mSAT-Hairpin linker | GgaaaatttagaaatgtttaatgtaggaCGtggaatatggcaagaaaactgaaaatcatgggaaatgagaaac  atccacttgtCGacttgaaaaatgaCGaaatcactaaaaaaCGtgaaaaatgagaaatgcacactgaaggN  TgggRTTatNNNNNNNNatgggRTTgNccttcagtgtgcatttctcatttttcaCGttttttagtgattt  CGtcatttttcaagtCGacaagtggatgtttctcattttttatgatttttagtttttttgtt | Sequencing |
