# Supplementary material for: Histone and DNA methylation control by H3 serine 10/threonine 11 phosphorylation in the mouse zygote
Source: Epigenetics Chromatin. 2017 Feb 14;10:5. doi: 10.1186/s13072-017-0112-x (PMC5307733; doi:10.1186/s13072-017-0112-x)
Supplement: Supplementary file 13 — Additional file 13. The list and detailed information about the antibodies used in this study. [file 13072_2017_112_MOESM13_ESM.doc]

**Antibodies used in this study**

| Name (Manufacturer, Catalog#, species origin) | Dilution | Applications |
| --- | --- | --- |
| Anti-H3K9me2 (gift from Thomas Jenuwein, rabbit polyclonal) | 1:1000 | IF/WB |
| Anti-H3K9me2 (Abcam, #ab1220, mouse monoclonal) | 1:1000 | WB |
| Anti-H3K9me3 (Millipore, #07-442, rabbit polyclonal) | 1:200/1:500 | IF/WB |
| Anti-H3S10phos (Cell Signaling, #3377, rabbit monoclonal) | 1:100 | IF |
| Anti-H3T11phos (Cell Signaling, #9767, rabbit monoclonal) | 1:100 | IF |
| Anti-G9a (Abcam, #ab31874, rabbit polyclonal) | 1:100 | IF |
| Anti-5mC (Calbiochem, #NA81, mouse monoclonal) | 1:1000 | IF |
| Anti-5hmC (Active motif, #39791, rabbit polyclonal) | 1:1000 | IF |
| Anti-5caC (Active motif, #61225, rabbit polyclonal) | 1:2000 | IF |
| Anti-ssDNA (Immuno-Biological Laboratories, #18731, rabbit polyclonal) | 1:400 | IF |
| Anti-eGFP (Antibodies-online GmbH, #AA1-246, goat polyclonal) | 1:2000 | IF/WB |
| Anti-mouse IgG Alexa Fluor® 647 (Life Technologies, #A-21237) | 1:200 | IF |
| Anti-rabbit IgG Rhodamine Red-X (Jackson ImmunoResearch Laboratories Inc., #83473) | 1:500 | IF |
| Donkey Anti-Goat IgG Antibody (Abcam, #ab6566) | 1:1000 | IF |
| Goat Anti-Mouse IgG Antibody, HRP conjugate (Millipore, #AP308P) | 1:5000 | WB |
| Goat Anti-Rabbit IgG Antibody, HRP-conjugate (Millipore, #12-348) | 1:2500 | WB |
| Donkey Anti-Goat IgG H&L, HRP conjugate (Abcam, #ab6885) | 1:5000 | WB |

Abbreviation: IF, immuno fluorescence; WB, western blotting;
